# ATP-triggered biomimetic deformations of bioinspired receptor-containing polymer assemblies†
†Electronic supplementary information (ESI) available. See DOI: 10.1039/c5sc00965k


**DOI:** 10.1039/c5sc00965k

**Published:** 2015-05-07

**Authors:** Qiang Yan, Yue Zhao

**Affiliations:** a Département de Chimie , Université de Sherbrooke , Sherbrooke , Québec , Canada J1K 2R1 . Email: Yue.Zhao@Usherbrooke.ca

## Abstract

Designing synthetic polymer assemblies that can sense a biological signal to mimic cell activities is elusive. We develop a class of block copolymer containing bioinspired host units as supramolecular catchers for the highly-selective capture of adenosine-5′-triphosphate (ATP). Driven by ATP, these block copolymers undergo a stepwise self-assembly and exhibit cascading deformation into highly-ordered nanostructures *via* the specific recognition effect between ATP and the receptor. By modulating the ATP concentration, one can precisely control the biomimetic evolution of these assemblies in diverse dimensionalities and geometries, like certain organellar deformations. Moreover, the ATP/polymer hybrid aggregates can be reversibly disassembled in response to phosphatase. The special ability of the artificial assemblies to sense intracellular bioactivators can offer new insight into bio-responsive nanomaterials for cellular applications.

## Abstract 2

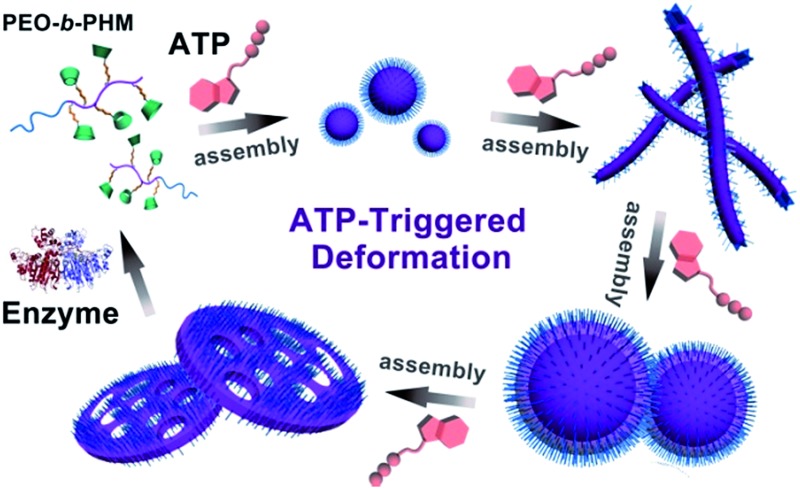
A block copolymer can recognize ATP through bioinspired receptors to initiate a series of deformation and morphological transitions of the polymer assemblies.

## Introduction

Responding to specific intracellular biological signals is one of the most inherent features of living organisms, and is crucial for maintaining cell activities, especially biological deformable motions.^1^ Controlled shape transformation, tuned by endogenous molecules, is ubiquitous in biological systems.^2^ Although they offer us many references,^3^ considering their componential multiplicity and self-organized complexity, it is very difficult to simulate these deformations *via* artificial molecular building blocks.^4^ Seeking new methodology to build molecular assemblies that can sense biomolecules and drive shape transformation is conducive to connecting self-assembly chemistry with biomimicry.

In this respect, stimuli-responsive polymeric assemblies have sparked considerable interest since they can respond to physical or chemical changes (*e.g.* pH, temperature, redox, gas, and light), and trigger a self-assembled shape transformation.^5^ To date, polymer stimuli-deformation is known to involve three fundamental principles: (i) the cleavage/linkage of chemical bonds, (ii) the configuration conversion of groups, and (iii) the phase transition of polymer chains.^6^ In contrast, the deformation of biological assemblies is usually mediated by a ligand–receptor effect, that is, a bioactivator (acting as a ligand) can be captured by a specific bioreceptor to tune the morphology of the biological assembly. However, almost all synthetic macromolecules have the limitation of being unable to bind such bioactivators.^7^ This is understandable since biological molecules are too intricate to be selectively recognized.^8^ Hence, designing novel polymers with bioinspired receptors capable of responding to cellular signals remains a tremendous challenge.^9^

ATP, as the cellular energy currency, is a central metabolite and critical biological signaling molecule, playing an irreplaceable role in many cell activities. Some nascent studies have made use of natural aptamers or chaperone proteins to prepare ATP-sensitive nanocarriers.^10^ In these systems, ATP molecules can manipulate the disassembly of these nanocarriers. Despite the progress on this frontier, synthetic polymers that can feedback ATP biosignals have not been developed so far. Thereby, exploiting ATP as an endogenous stimulus to subtly modulate the dynamic behavior of polymer assemblies holds promise for biomimicry.

**Fig. 1:**
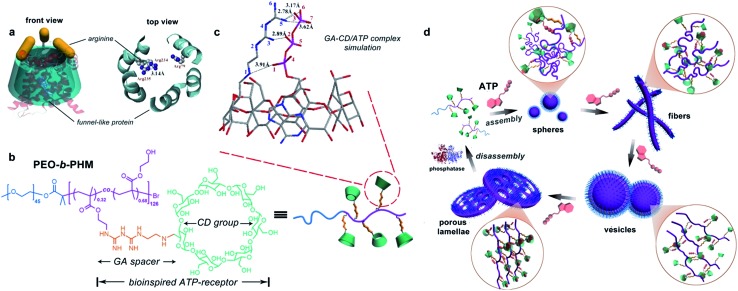
(a) Natural ATP carrier protein: front (left) and top view (right) of its funnel-like cavity and three arginine lids. (b) Chemical structure of the bioinspired receptor-containing block copolymer (PEO-*b*-PHM): its side chain has a large number of mimetic ATP-capturers composed of a biguanidine (GA) spacer and a β-cyclodextrin (CD) host moiety. (c) Molecular docking results of GA–CD and ATP ligand–receptor complexation (calculated by software SYSBL 7.3 methods, in which the color sticks represent: nitrogen (blue); carbon (grey); oxygen (red) and phosphorus (purple). All hydrogen atoms are omitted for clarity). (d) ATP-triggered self-assembly and morphological transformation of these bioinspired receptor-containing block copolymers to biomimic the organellar deformations.

Natural ATP carrier protein has a funnel-like structure (∼20 Å in length and 8 Å in diameter) capped by arginine lids (Fig. 1a).^11^ It can specifically recognize one ATP molecule by host–guest chemistry and H-bonding interactions. Inspired by its structural features, here we have delicately designed a special biomimetic receptor-containing diblock copolymer. The artificial groups can seize ATP bioactivators by specific ligand–receptor interactions, leading to the formation of ATP/polymer complexes, which can further induce polymer self-assembly. Moreover, owing to the multiple receptors attached on the main chain, the polymer can quantitatively sense the amount of ATP. This offers the chance to achieve ATP-responsive cascading deformations from primary assemblies to sophisticated structures, mimicking biological membrane remodelling (Fig. 1d).

## Results and discussion

### Designing a synthetic ATP-receptor

To realize our goal, we targeted a kind of water-soluble polymer with bioinspired receptors capable of trapping ATP such that the ATP/polymer complexes could be readily formed. The ability of each independent polymer to capture the ATP is essential, as this has a profound effect not only on tuning the number of ligand–receptor complementary pairs but also on controlling their self-assembling nanostructures. We therefore synthesized a diblock copolymer, PEO_45_-*b*-PHM_126_, *via* atom transfer radical polymerization.^12^ As shown in Fig. 1b, PEO is a biocompatible poly(ethylene oxide) block (*M*_n,PEO_ = 2.0 kDa) while PHM is a functionalized host-abundant polymethacrylate block (*M*_n,PHM_ = 66.5 kDa) in which β-cyclodextrin (CD), serving as macrocyclic pendants, is randomly grafted onto the backbone bridged by a short biguanidine (GA) spacer (32% grafting density, each chain has ∼40 pendants).^13^ We expect that such a pocket-like GA–CD functionality can act as a receptor to catch ATP because the CD moiety (7.8 Å cavity diameter), simulating the ATP carrier's funnel-like structure, could bind the nucleotide component of ATP *via* a host–guest interaction and the GA spacer, analogous to the arginine caps, could stabilize the triphosphate component of ATP *via* H-bonding.

We first aimed to survey whether this bioinspired receptor could seize the ATP bioactivator. A molecular docking simulation was used to study the interactions between the GA–CD model compound and ATP.^14^ Based on the prediction of the noncovalent distances, the GA moiety theoretically forms a quintuple H-bonding network with the ATP triphosphate tail (N5–O3, 2.78 Å; N5–O6, 3.17 Å; N5–O7, 3.62 Å; N3–O2, 2.89 Å; N1–O1, 3.91 Å), and the CD moiety can envelop the nucleotide head group tightly (Fig. 1c). These results imply that the GA–CD group has the possibility to be an efficient ATP-receptor.

### Specific recognition of ATP by the polymeric receptor

**Fig. 2:**
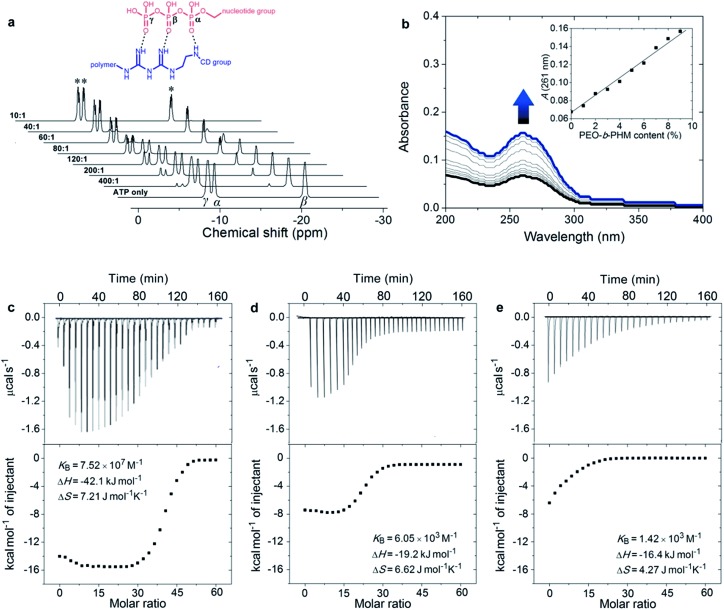
ATP/polymer ligand–receptor interactions: (a) ^31^P NMR titration of ATP (40 mM) with PEO-*b*-PHM at variable concentration ratios (D_2_O). The inset shows the H-bonding interaction sites between ATP's triphosphate region and the copolymer's GA species. (b) UV-vis absorption changes of ATP (0.1 mM) upon addition of PEO-*b*-PHM (0–10% content). The inset gives the absorption variation (261 nm) as a function of copolymer content. (c–e) ITC results show the three ligand–receptor interactions: ATP/PEO-*b*-PHM (c), ATP/C_1_ (C_1_ is a copolymer counterpart without the GA spacer) (d), and ATP/C_2_ (C_2_ is another copolymer counterpart without the CD pendant) (e). 60 μM ATP solution was injected into 1.0 μM copolymer solution at 293 K (all the experiments were in Tris–HCl buffer, pH = 7.20).

According to the semiempirical simulation, our synthetic polymer can strongly bind to ATP as a result of the synergism between the H-bonding of the GA/triphosphate group and the host–guest chemistry of the CD/nucleotide group. To further elucidate the ATP/PEO-*b*-PHM ligand–receptor recognition, we employed ^31^P NMR methodology and UV-vis spectroscopy to respectively monitor the two classes of supramolecular interactions. In the absence of the copolymer, ATP showed three phosphorus peaks at *δ* = –9.5 (γ-P), –10.6 (α-P), and –21.4 (β-P) ppm. Upon injecting increasing amounts of the copolymer into ATP in a molar ratio from 1 : 400 to 1 : 10, the three initial signals were gradually weakened, accompanied by another set of broad signals of increasing intensity at *δ* = –7.6 (d, ^3^ *J*_β,γ_ = 11.4 Hz), –8.4 (d, ^3^ *J*_α,β_ = 16.5 Hz) and –19.1 (dd, ^3^ *J* = 16.5, 11.4 Hz) ppm, indicating that the amount of free ATP was slowly decreasing and a new ATP complex was being generated (Fig. 2a). The shifts to low field and the spin–spin splitting can be ascribed to the formation of multivalent interactions between the GA moiety and the phosphate region.^15^ On the other hand, UV-vis spectra further disclosed the host–guest interaction. Without the copolymer, the ATP solution displayed a typical absorption for adenosine at 261 nm;^16^ However, upon gradual addition of PEO-*b*-PHM, this absorption showed a doubling of intensity from 0.07 to 0.16, indicating that the adenosine species was bound to the cavity of CD to form an inclusion complex (Fig. 2b). This host-enhanced UV-absorption can be observed in other host–guest systems.^17^ In addition, monitoring the proton shifts of ATP and the nucleotide region by ^1^H NMR and 2D NOSEY spectra confirmed this result (Fig. S7 in the ESI†). The average of the stoichiometric binding ratio between ATP and one polymer is measured to be 36 by a Job's plot experiment (Fig. S8 in ESI†), which is close to the copolymer grafting density (∼40). It means that this polyvalent receptor can not only recognize the ATP but also respond to the amount of ATP in an almost quantitative way.

Next we aimed to understand how strong the ATP/PEO-*b*-PHM interaction was. Here we used an isothermal titration calorimetry (ITC) method to resolve this problem. ITC is a quantitative technique that can directly measure biological intermolecular interactions and thermodynamic parameters.^18^ An ATP stock solution (60 μM) was injected into the PEO-*b*-PHM solution (1.0 μM, Tris–HCl buffer, pH = 7.20). The binding affinity (*K*_B_) was found to be 7.52 × 10^7^ M^–1^, which is the most approximate data to that of the natural ATP carrier so far. The binding stoichiometry, *n*, fits to 34.8, which is consistent with the UV-vis results. The association enthalpy and entropy are Δ*H* = –42.1 kJ mol^–1^ and Δ*S* = 7.21 J mol^–1^ K^–1^, respectively, and the Gibbs free energy (Δ*G*) was calculated to be –44.2 kJ mol^–1^ (Fig. 2c; Table S1 in ESI†). Since the total binding affinity arises from the H-bonding and host–guest combination, we prepared two copolymer counterparts to unravel the independent contribution of the two noncovalent forces. The two counterparts, referred to as C_1_ and C_2_, lack the GA spacer and CD pendant, respectively (Fig. S6 in ESI†). In control experiments, the association constants and free energies were *K*_B_ = 6.05 × 10^3^ M^–1^ and Δ*G* = –21.2 kJ mol^–1^ for ATP/C_1_, and *K*_B_ = 1.42 × 10^3^ M^–1^ and Δ*G* = –17.7 kJ mol^–1^ for ATP/C_2_. The above two binary systems exhibited much weaker association than the ATP/PEO-*b*-PHM complex (Fig. 2d and e; Table S1 in ESI†), pointing out that a polymer possessing only an individual supramolecular force is insufficient to recognize ATP biomolecules. Furthermore, on the basis of the energy terms, the independent contributions of the host–guest interaction and the H-bonding are 48% and 40%, respectively. It is worth noting that the extra binding energy (accounting for 12%) results from a positive cooperative effect of the GA and CD moieties (Fig. S9 in ESI†).

**Fig. 3:**
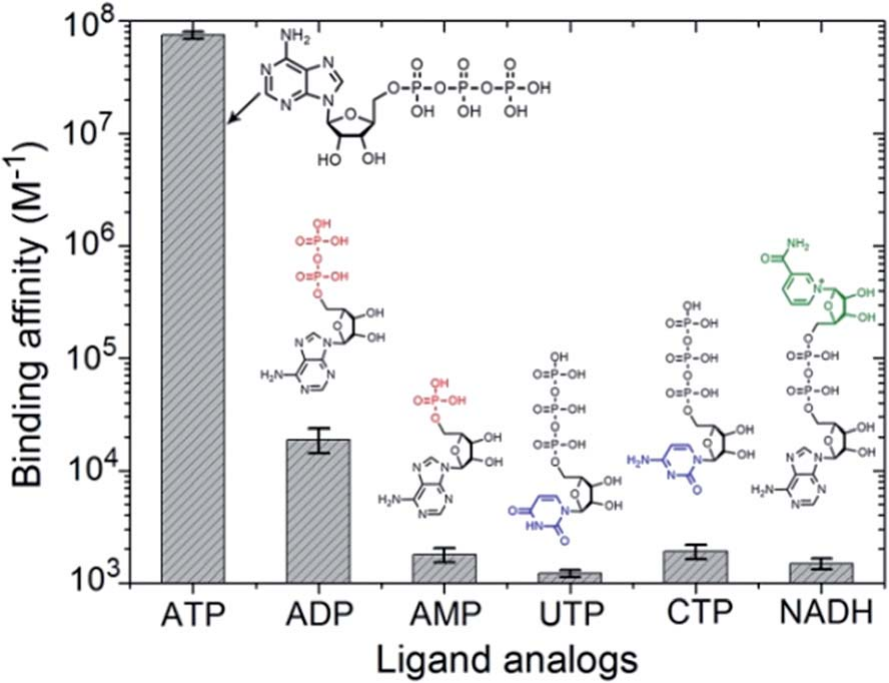
The specific recognition between PEO-*b*-PHM copolymer and ATP. The ATP/PEO-*b*-PHM complex shows the highest binding affinity of ∼10^7^ M^–1^. Other ATP analogues (ADP, AMP, UTP, CTP and NADH) exhibit much lower binding affinities (∼10^3^ M^–1^). All experiments were performed using ITC (adding 60 μM of ATP into 1.0 μM copolymer solution at 293 K in Tris–HCl buffer, pH = 7.20).

In the intracellular environment, there are some other biological metabolites with a similar structure to ATP. Hence we wondered whether our polymer receptor had high specificity and selectivity to ATP. As shown in Fig. 3, indeed ATP has by far the strongest binding affinity with PEO-*b*-PHM (>10^7^ M^–1^). Considering its similarities, adenosine-5′-diphosphate (ADP), due to the lack of a phosphate group for the formation of multiple H-bonds, is unable to form a stable ligand–receptor complex, as indicated by the dramatically decreased binding affinity of *K*_B_ = 1.9 × 10^4^ M^–1^. In the case of adenosine-5′-monophosphate (AMP), it cannot even associate with the polymer. Uridine-5′-triphosphate (UTP) and cytidine-5′-triphosphate (CTP) are two other interferents. Even though they have the capability to form multiple H-bonds, their binding affinities are less than ∼10^3^ M^–1^. The reason for this is that the sizes of their nucleotide head groups mismatch the CD cavity. Finally, for nicotinamide adenine dinucleotide (NADH), its bulky end-group hinders the formation of an inclusion complex due to a steric effect. These results further demonstrate the high specificity for ATP of our bioinspired receptor.

### ATP-driven polymer self-assembly and shape transformation

**Fig. 4:**
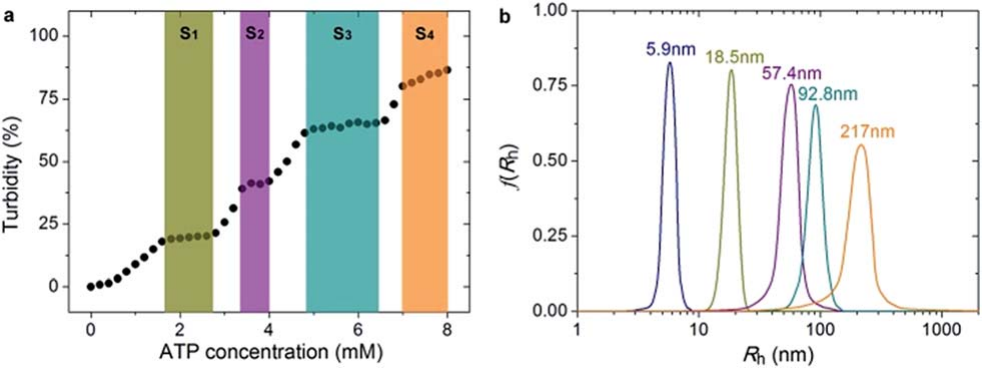
(a) Turbidity changes show the four stable self-assembly phase states (S1–S4, coloured regions) and the intermediate states (blank) of the PEO-*b*-PHM assemblies under different ATP levels. (b) DLS results show the size changes of the polymer assemblies in various ATP levels: navy, 0 mM; dark yellow, 1.8 mM; purple, 3.6 mM; cyan, 5.0 mM and orange, 8.0 mM (from left to right).

Since PEO-*b*-PHM can specifically recognize ATP bioactivators, we wanted to further investigate whether the polymer self-assembly and shape transformation could be driven by ATP. PEO-*b*-PHM is water-soluble, forming a homogenous and transparent solution (∼0.42 mM maximum solubility). All the experiments fixed the copolymer concentration at 0.20 mM (in buffer, with pH = 7.20) and varied the ATP stimulus from 0 mM to 8 mM, which matched well with the intracellular ATP concentration (1–10 mM).^19^ As we added ATP into the copolymer solution, interestingly, it became increasingly opaque, implying a self-assembled colloid had formed (Fig. S10 in ESI†). Since the size of a colloidal particle has a positive correlation with solution turbidity, an ATP concentration-resolved transmittance experiment was carried out to detect the particle size change.^20^ The work profile covered four fast-ascent stages and four platform stages (Fig. 4a): In the range of 0–1.6, 2.8–3.4, 4.0–4.8, and 6.4–7.0 mM of ATP, the turbidity rose rapidly from 0% → 18%, 20% → 39%, 42% → 61%, and 65% → 79%, respectively, meaning that the sizes of the ATP/polymer complexes underwent a stepped increase. In contrast, every plateau (S_1_–S_4_) between two adjacent climbing stages (1.6–2.8, 3.4–4.0, 4.8–6.4, and 7.0–8.0 mM of ATP, coloured regions in Fig. 4a) suggested that the variation in micellar dimension was negligible and, probably, corresponded to a certain stable phase. Similar results can be found in another stimuli-responsive systems.^4*b*^ Dynamic light scattering (DLS) further supported this result (Fig. 4b). By slowly adding ATP to a given copolymer solution, in the four platform periods, the average hydrodynamic radius, *R*_h_, showed a continuous growth from an initial value of 5.9 nm (no ATP) to 18.5 nm (1.8 mM ATP), 57.4 nm (3.6 mM ATP), 92.8 nm (5.0 mM ATP) and 217 nm (8.0 mM ATP). Such a series of size changes reveals that the morphological differentiation of the ATP/polymer complex relies on the ATP stimulation levels.

**Fig. 5:**
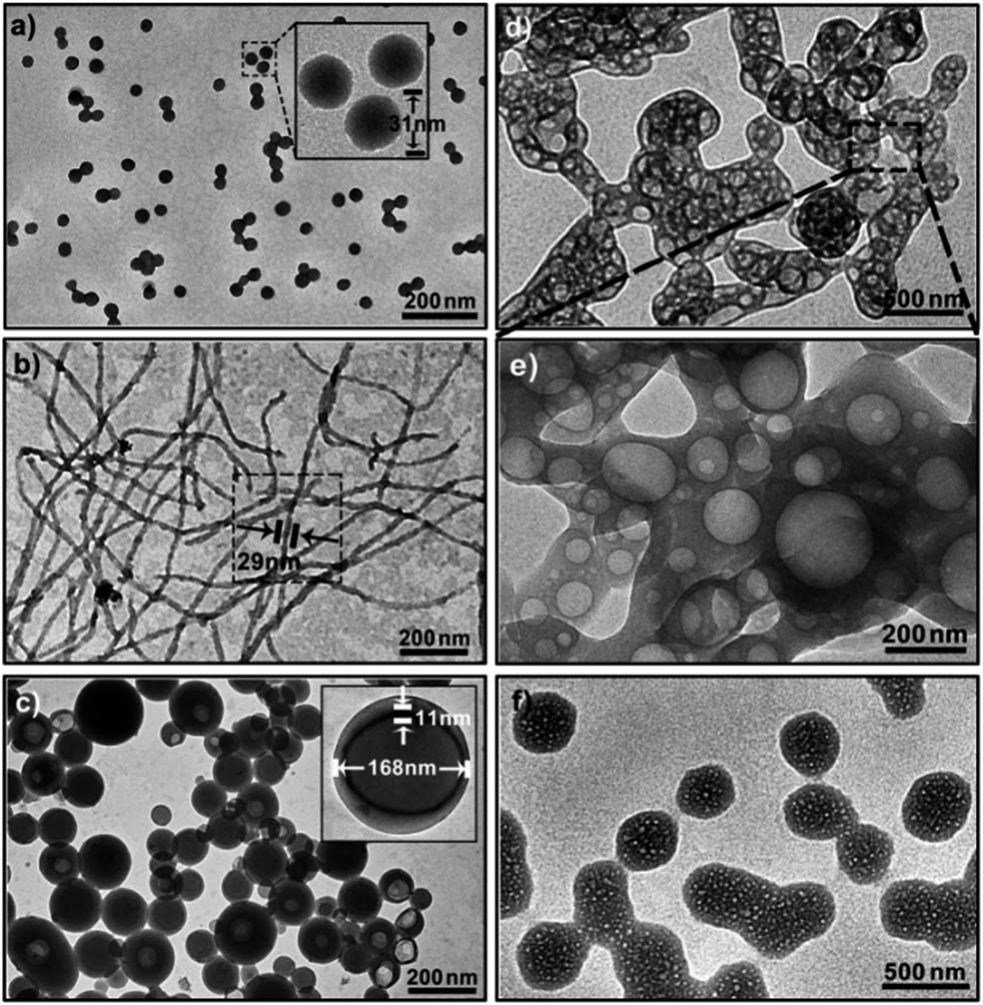
TEM images of the shape transformation of the polymer assemblies under different ATP levels: (a) spherical micelles with 1.8 mM ATP, (b) worm-like micelles with 3.6 mM ATP, (c) vesicles with 5.0 mM ATP, (d) fused vesicular networks with 7.2 mM ATP, (e) the magnified view for the sponge-like nanopores, and (f) porous interconnected lamellae with 8.0 mM ATP. The polymer concentration was fixed at 0.20 mM in Tris–HCl buffer, pH = 7.20.

To visualize and track the deformation details at the various stages, we used transmission electron microscopy (TEM). With the aid of a little ATP (1.8 mM), the copolymers could self-assemble into near-monodisperse spherical micelles. They were typical corona-core structures with a homogenous diameter of 31 ± 2 nm determined by TEM auto-statistics over 250 particles (Fig. 5a and inset; Fig. S11 in ESI†), which is in accordance with the DLS results (*R*_h_ = 18.5 nm). Furthermore, the gyration radii, *R*_g_, of these aggregates were ∼14.6 nm determined by static light scattering (SLS). The shape factor, *ρ* = *R*_g_/*R*_h_, was used as a sensitive parameter to identify the geometry of the polymer aggregates.^21^ In this case, the *ρ* value was calculated to be 0.789, corresponding to the theoretical value of a solid sphere (*ρ*_T_ = 0.774, Fig. S12 in ESI†). After increasing the ATP concentration to 3.6 mM, these spheres completely vanished, and instead, a great number of worm-like micelles appeared in the solution (Fig. 5b; Fig. S13 in ESI†). These nanofibers were hundreds of nanometers in length and had an almost uniform diameter of 29 ± 3 nm. Meanwhile, the *ρ* value was 1.893, further ascertaining their fibrous structure (*ρ*_T_ = 1.732 for cylinders; Fig. S14 in ESI†). In view of their similar diameter to spherical micelles, they were possibly formed by the conjunction and remodelling of multiple spheres. An intermediate state with the appearance of a string of beads upon the addition of 2.8 mM of ATP confirmed the process of linear micellar connection (Fig. S15 in ESI†). When the ATP concentration reached 5.0 mM, the dominant population transformed to large vesicular nanostructures (Fig. 5c; Fig. S16 in ESI†), as confirmed by a *ρ* of 1.082 (*ρ*_T_ ∼ 1 for hollow spheres; Fig. S17 in ESI†). The size of these aggregates ranged from 55 nm to 194 nm (average ∼168 nm) and the wall thickness was about 11 nm. Note the tendency of the vesicles to cohere to each other *via* the attachment of their membranes. Interestingly, further increasing ATP strength (7.2 mM) led to a broad fusion among different vesicles to produce a network structure, like a large porous sponge (Fig. 5d; Fig. S18 in ESI†). In particular, the nanopore size was in line with that of the vesicles (Fig. 5e), proving the fusion mechanism. Afterwards, the sponge-like objects were self-flattened, and finally adopted a kind of irregular interconnected lamellar architecture upon the addition of 8.0 mM ATP (Fig. 5f; Fig. S19 in ESI†). Their average scale exceeded ∼500 nm and the nanopore diameter fell to 20–40 nm, indicating that these pores were compressed strongly in the flattening process. This continuous shape evolution (nanospheres → nanofibers → vesicles → porous lamellae) is in many ways reminiscent of biological deformations.

### ATP-triggered deformation mechanism

This striking biomimetic polymer shape transformation depends on amphiphilic alterations to the block copolymer. In the absence of ATP, PEO-*b*-PHM can be dissolved in aqueous solution. When ATP is added, it is trapped by the polymer receptors to form ATP/polymer complexes. With increasing ATP concentration, the number of complexed units gradually increases, resulting in a continuous molecular weight augmentation, as revealed by gel permeation chromatography (from 68.5 kDa at 0 mM ATP to 84.1 kDa at 8.0 mM ATP; Fig. S20 in ESI†). Since the complexation segments change from hydrophilic to hydrophobic while the non-complexed portions remain hydrophilic, the shift in the hydrophilic–hydrophobic balance becomes a driving force that influences the polymer self-assembly. The increase in the amount of ATP causes a gradual decrease in the hydrophilic block volume fraction (*f*). It is known that *f* is related to the geometry of block copolymer amphiphiles. Theoretically, spheres should be formed when *f* > 50%, worm-like micelles when 40% < *f* < 50%, vesicles for 25% < *f* < 40%, and other complex lamellar structures for *f* < 25%.^22^ In the case of PEO-*b*-PHM (0.20 mM), by increasing the ATP concentration, the *f* values show a clear reduction from 77% (1.8 mM ATP) to 52% (3.6 mM ATP) to 31% (5.0 mM ATP) and finally to 12% (8.0 mM ATP), corresponding to the globular, fibrous, vesicular and lamellar structures, respectively (Table S2 in ESI†).

### The specificity of ATP-driven biomimetic deformations

To be better suited for application in a cellular environment, it is desirable for this biomimetic deformation to have ATP specificity and selectivity. From the above experiments, we know that polymer assembly induces an increase in the solution turbidity. Based on this characteristic, we surveyed the effects of some ATP analogues (Fig. S21 in ESI†). Regarding the maximum turbidity change of the ATP/polymer as a reference standard, ADP only caused a 27% turbidity change, even at a higher concentration (20 mM). This indicated that it was difficult for ADP to form available ADP/polymer complexes for further self-assembly. Moreover, because of the much weaker binding affinity to PEO-*b*-PHM, AMP, UTP, CTP and NADH had no capability to provoke similar assembling. This direct evidence was also corroborated by the TEM images (Fig. S22 in ESI†). These findings indicate that ATP is the only biosignal that can efficiently activate the polymeric shape transformation.

### Enzyme-responsive disassembly

**Fig. 6:**
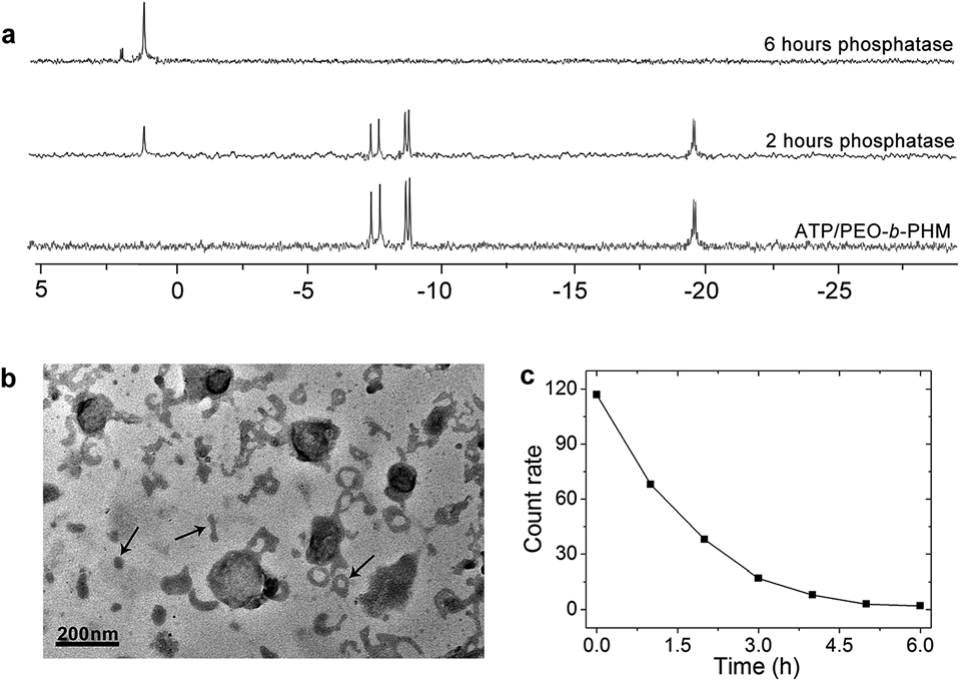
(a) ^31^P NMR spectra of ATP/PEO-*b*-PHM assemblies at various times upon addition of phosphatase. (b) TEM image of the intermediate state of the lamellar disassembly after enzyme treatment for 2 h. (c) DLS count rate data of the ATP/PEO-*b*-PHM complex after treatment with phosphatase for 6 h. The polymer assemblies were kept in Tris–HCl buffer, pH = 7.20.

Ultimately, we expected that the ATP/polymer hybrid aggregates could be disassembled under a biological stimulus. Phosphatase is a type of enzyme that can hydrolyze ATP into free phosphate groups. A small quantity of phosphatase (120 U L^–1^), which is comparable with the average amount of phosphatase present in a healthy adult, was injected into the ATP/polymer assemblies (lamellae). ^31^P NMR gave evidence of enzymatic disruption of the complex. Fig. 6a shows that the complexed ATP peaks (–7.6, –8.4 and –19.1 ppm) are depressed by 45% and that a new peak at 1.2 ppm, ascribed to free phosphoric acid, appeared upon phosphatase treatment for 2 h. After 6 h, only a phosphoric acid peak could be observed, indicating the entire dissociation of these ATP/polymer assemblies. Since the poly“complex” could respond to the enzyme, we considered that this feature could be inherent to their self-assembled structure. Indeed, after treating with enzyme for 2 h, TEM disclosed the intermediate state of the disassembling lamellae. As shown in Fig. 6b, it is clear that the area of these lamellae was significantly reduced from 0.5 μm down to smaller than 200 nm and that a large number of nanofragments had appeared in the solution (arrows), which indicates partial dissociation of these lamellar objects. Finally, these polymer aggregates were completely disassembled and vanished after long-term enzyme treatment (Fig. S23 in ESI†). The count rate data from DLS also confirmed that all the aggregates disappeared after about 6 h (Fig. 6c).

## Conclusions

In summary, we have developed a new kind of ATP-responsive block copolymer conjugated to bioinspired ATP-receptors. These synthetic macromolecules exhibit a strong binding affinity (>10^7^ M^–1^), and high specificity and selectivity to the ATP bioactivator. By capturing ATP, the polymer can form ATP/polymer hybrid complexes. One can modulate ATP stimulation levels to precisely control the self-assembly architecture with anticipated geometries, dimensionalities and shape transformable behaviors. Reversible disassembly is the response to a phosphatase. We envisage that this bio-responsive polymer model will open up an avenue to take advantage of synthetic macromolecules to build smart biomimetic assemblies for imitating cellular and organellar activities.
