# Supplementary material for: ATP-triggered biomimetic deformations of bioinspired receptor-containing polymer assemblies
Source: Chem Sci. 2015 May 7;6(7):4343–9. doi: 10.1039/c5sc00965k (PMC5707515; doi:10.1039/c5sc00965k)
Supplement: Supplementary file 1 [file SC-006-C5SC00965K-s001.pdf]

Electronic Supplementary Material (ESI) for Chemical Science.
This journal is © The Royal Society of Chemistry 2015

Supplementary Information

ATP-triggered biomimetic deformations of bioinspired receptor-containing polymer assemblies

Qiang Yan and Yue Zhao*

Département de Chimie, Univeristé de Sherbrooke, Sherbrooke, Quebec, Canada J1K 2R1.
Corresponding Author to Prof. Yue Zhao. (Email:Yue.Zhao@Usherbrooke.ca).

1. Polymer Synthetic Routes

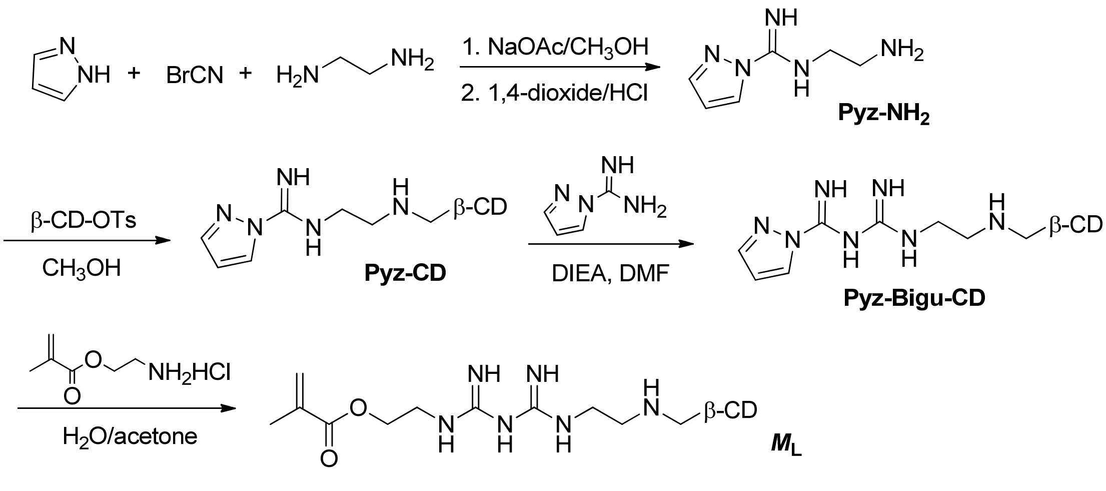

Fig. S1. The synthetic procedure of specific monomer (***M***$_L$) with bioinspired receptor unit

Synthesis of Pyz-NH$_2$. Synthesis of Pyz-NH$_2$ was on the basis of previous literature as follows[1]: Ethylenediamide (1.80 g, 30.0 mmol) was treated with cyanogens bromide (2.11 g, 20.0 mmol) and sodium acetate (1.64 g, 20.0 mmol) and then dissolved in dry methanol (20 ml). The reaction mixture was stirred overnight in room temperature to afford crude cyanamide solid. Subsequent condensation of cyanamide with pyrazole (1.70 g, 25.0 mmol) in 4 M HCl/1,4-dioxane (20 ml) reacted for 12 h and gave the crude products. Finally, the slight yellow solid was purified by recrystallization twice in methanol/H_2O mixture (4/1, v/v) to afford Pyz-NH$_2$ (2.42 g, 79% yield). ^{1}H NMR (300 MHz, DMSO-d_6): δ_H (ppm) = 8.35–8.39 (m, 3H, in pyrazole group), 6.45 (s, 1H, -C(=N***H***)-NH-), 2.93 (d, 2H, -NHC***H$_2$***CH$_2$NH$_2$), 2.42 (d, 2H, -NHCH$_2$C***H$_2$***NH$_2$), 1.62–1.90 (br, 2H, -NHCH$_2$CH$_2$N***H$_2$***). ^{13}C NMR (300 MHz, DMSO-d_6): δ_C (ppm) = 151.6 (N-***C***(=NH)-NH in guanidine), 140.8 (-***C***H=N-N- in pyrazole), 130.6 (-CH=***C***H-N-N= in pyrazole), 108.3 (=CH-***C***H= in pyrazole), 53.3 (-NH-CH$_2$-***C***H$_2$-NH$_2$), 38.5

(-NH-$\boldsymbol{C}H_2$-CH_2-NH_2). FT-IR (ν, KBr, cm^{-1}): 3480 (-NH_2), 2945 (-CH_2-), 1667 (-C=NH), 1552 (-C=C- in pyrazole). ESI-MS: calcd. for $C_6H_{11}N_5$ $[M+H]^+$: m/z = 154.10; found: 154.13.

Synthesis of Pyz-CD. The mono-6-deoxy-6-(p-tolysulfonyl)-β-CD (β-CD-OTs) was prepared according to the previous literature.[2] β-CD-OTs (3.40 g, 2.6 mmol) was first dissolved in dry methanol and then reacted with an excess amount of Pyz-NH_2 (2.02 g, 13.0 mmol) at 80 °C for 8 h. After the reaction finished, the solution was cooled down and most of solvent was removed by rotary evaporation. The crude product was then dissolved in dimethyl sulfoxide/H_2O mixture (1/1, v/v) and precipitated in acetone for three times. The product Pyz-CD was dried for overnight in a vacuum oven (2.47 g, 65% yield). ^1H NMR (300 MHz, DMSO-d_6): δ_H (ppm) = 8.35—8.39 (m, 3H, pyrazole group), 6.48 (s, 1H, -C(=N***H***)-NH-), 5.65—5.79 (m, 14H, OH-2,3 in CD), 4.81 (d, 7H, H-1 in CD), 4.40—4.52 (m, 5H, OH-6 in CD), 3.52—3.75 (m, 28H, H-3,5,6 in CD), 3.23—3.45 (m, 14H, H-2,4 in CD), 2.79 (d, 2H, -NHC$\boldsymbol{H_2}$$CH_2$$NH_2$), 2.42 (d, 2H, -NH$CH_2C\boldsymbol{H_2}$$NH_2$). ^{13}C NMR (300 MHz, DMSO-d_6): δ_C (ppm) = 151.2 (N-***C***(=NH)-NH in guanidine), 139.6 (-***C***H=N-N- in pyrazole), 130.1 (-CH=***C***H-N-N= in pyrazole), 108.5 (=CH-***C***H= in pyrazole), 102.6, 81.9, 73.9, 72.8, 72.6, 63.1 (C1-C6 in CD), 54.9 (-NH-CH_2-$\boldsymbol{C}H_2$-NH_2), 38.8 (-NH-$\boldsymbol{C}H_2$-CH_2-NH_2). FT-IR (ν, KBr, cm^{-1}): 3520 (m, O-H), 3467 (br, N-H), 2940 (s, -CH_2-), 1647 (s, -C=NH), 1552 (m, -C=C- in pyrazole), 1151 (s, C-O). MALDI-TOF-MS (dithranol): calcd. for $C_{48}H_{79}N_5O_{34}$ $[M]^+$: m/z = 1269.46; found: 1269.11.

Synthesis of Pyz-Bigu-CD. This synthetic procedure was according to the previous literature as follows[3]: 1*H*-Pyrazole-1-carboxamidine hydrochloride (0.22 g, 2.0 mmol), Pyz-CD (2.05 g, 1.6 mmol) were dissolved in 1 ml of DMF and added the diisopropyl ethylamine (DIEA, 0.26 g, 2.0 mmol). Then the mixture was stirred for three days at room temperature. Diethyl ether (15 ml) was added and the resulting sticky solid was collected, washed with diethyl ether and dried in vacuum oven to yield crude product. Recrystallization of the crude product from ethanol/diethyl ether (3/1, v/v) yielded a white product (Pyz-Bigu-CD, 1.06 g, 47% yield). ^1H NMR (300 MHz, DMSO-d_6): δ_H (ppm) = 8.39 (m, 3H, pyrazole group), 7.85 (s, 1H, pyrazole-C(=N***H***)-NH-), 6.79 (s, 1H, pyrazole-C(=NH)-NH-C(=N***H***)-NH-), 5.65—5.79 (m, 14H, OH-2,3 in CD), 4.81 (d, 7H, H-1 in CD), 4.40—4.52 (m, 5H, OH-6 in CD), 3.52—3.75 (m, 28H, H-3,5,6 in CD), 3.23—3.45 (m, 14H, H-2,4 in CD), 2.87 (d, 2H, -NHC$\boldsymbol{H_2}$$CH_2$$NH_2$), 2.51 (d, 2H, -NH$CH_2C\boldsymbol{H_2}$$NH_2$). ^{13}C NMR (300 MHz, DMSO-d_6): δ_C (ppm) = 155.6, 151.2 (-***C***(=NH)-NH in biguanidine), 141.1 (-***C***H=N-N- in pyrazole), 130.1 (-CH=***C***H-N-N= in pyrazole), 109.0 (=CH-***C***H= in pyrazole), 102.6, 81.9, 73.9, 72.8, 72.6, 63.1 (C1-C6 in CD), 54.9 (-NH-CH_2-$\boldsymbol{C}H_2$-NH_2), 36.8 (-NH-$\boldsymbol{C}H_2$-CH_2-NH_2). FT-IR (ν, KBr, cm^{-1}): 3520 (m, O-H), 3447 (br, N-H), 2940 (s, -CH_2-), 1647 and 1629 (s, -C=NH), 1550 (m, -C=C- in pyrazole), 1149 (s, C-O). MALDI-TOF-MS (dithranol): calcd. for $C_{49}H_{81}N_7O_{34}$ $[M]^+$: m/z = 1311.48; found: 1311.92.

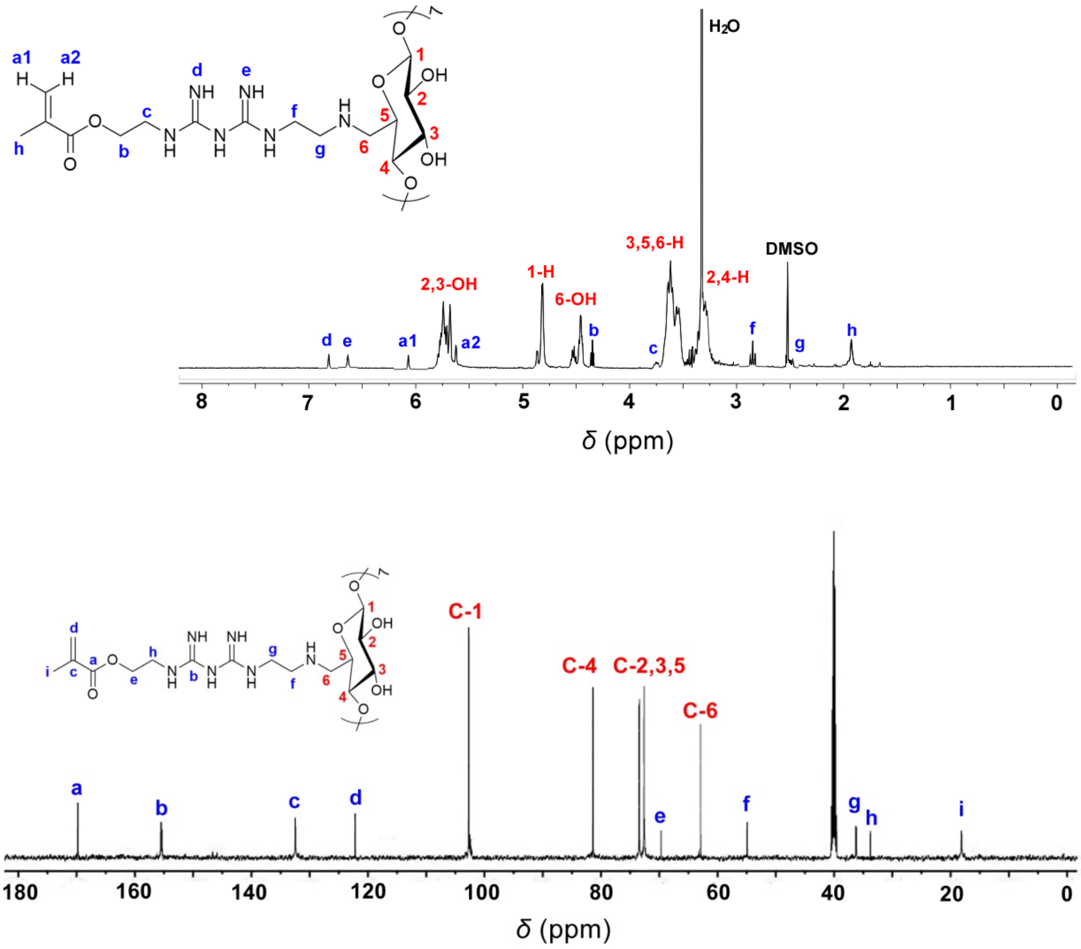

Fig. S2. ^{1}H NMR (top) and ^{13}C NMR (down) spectra of the specific monomer ($\boldsymbol{M}_{\mathrm{L}}$)

Synthesis of the specific monomer $\boldsymbol{M}_{\mathrm{L}}$ with a biguanidine spacer and CD pendant. The above product was often used for the guanylation process by introducing primary amine.[3,4] Typically, a solution of 2-aminoethyl methacrylate hydrochloride (0.17 g, 1.0 mmol), Pyz-Bigu-CD (1.04 g, 0.8 mmol), DIEA (0.13 g, 1.0 mmol) and 4 ml of H_2O/acetone (1/1, v/v) was stirred for 4 h at room temperature and then was heated to 50 ^{o}C for 12 h. The reaction mixture was extracted three times with 10 ml ether and the aqueous phase lyophilized to afford a crude product which was recrystallized from methanol/ether mixture (4/1, v/v) to give a functionalized monomer $\boldsymbol{M}_{\mathrm{L}}$ (0.79 g, 68% yield). ^{1}H NMR (300 MHz, DMSO-d_6): δ_{H} (ppm) = 6.80 (s, 1H, -CH_2NH-C(=N$\boldsymbol{H}$)-), 6.65 (s, 1H, -NH-C(=NH)NHC(=N$\boldsymbol{H}$)-NH-), 6.04 (s, 1H, C$\boldsymbol{H_2}$=CH(CH_3)COO-), 5.58 (s, 1H, C$\boldsymbol{H_2}$=CH(CH_3)COO-), 5.65─5.79 (m, 14H, OH-2,3 in CD), 4.81 (d, 7H, H-1 in CD), 4.43─4.58 (m, 5H, OH-6 in CD), 4.30 (t, 2H, -COOC$\boldsymbol{H_2}$$CH_2$NH-), 3.52─3.75 (m, -COO$CH_2C\boldsymbol{H_2}$NH- and H-3,5,6 in CD), 3.23─3.45 (m, 14H, H-2,4 in CD), 2.82 (d, 2H, -NHC$\boldsymbol{H_2}$$CH_2$NH-), 2.54 (d, 2H, -NH$CH_2C\boldsymbol{H_2}$NH-), 1.94 (s, 3H, -C$\boldsymbol{H_3}$). ^{13}C NMR (300 MHz, DMSO-d_6): δ_{C} (ppm) = 169.9 (-$\boldsymbol{C}$OOCH_2-), 155.6, 155.1 (-$\boldsymbol{C}$(=NH)-NH in biguanidine), 132.5 (CH_2=$\boldsymbol{C}$(CH_3)-), 124.8 ($\boldsymbol{C}$$H_2$=C($CH_3$)-), 102.6, 81.9, 73.9, 72.8, 72.6, 63.1 (C1-C6 in CD), 69.5 (-COO$\boldsymbol{C}$$H_2$$CH_2$-), 54.9

(-NH-CH$_2$-***C***H$_2$-NH$_2$), 36.4 (-NH-***C***H$_2$-CH$_2$-NH$_2$), 34.1 (-COOCH$_2$***C***H$_2$-), 18.1 (CH$_2$=C(***C***H$_3$)-). FT-IR (ν, KBr, cm^{-1}): 3520 (m, O-H), 3457 (br, N-H), 2942 (s, -CH$_2$-), 1710 (s, C=O), 1669 (m, C=C), 1627 (s, -C=NH), 1149 (s, C-O). MALDI-TOF-MS (dithranol): calcd. for $C_{52}H_{88}N_6O_{36}$ [M]$^+$: *m/z* = 1372.52; found: 1372.19.

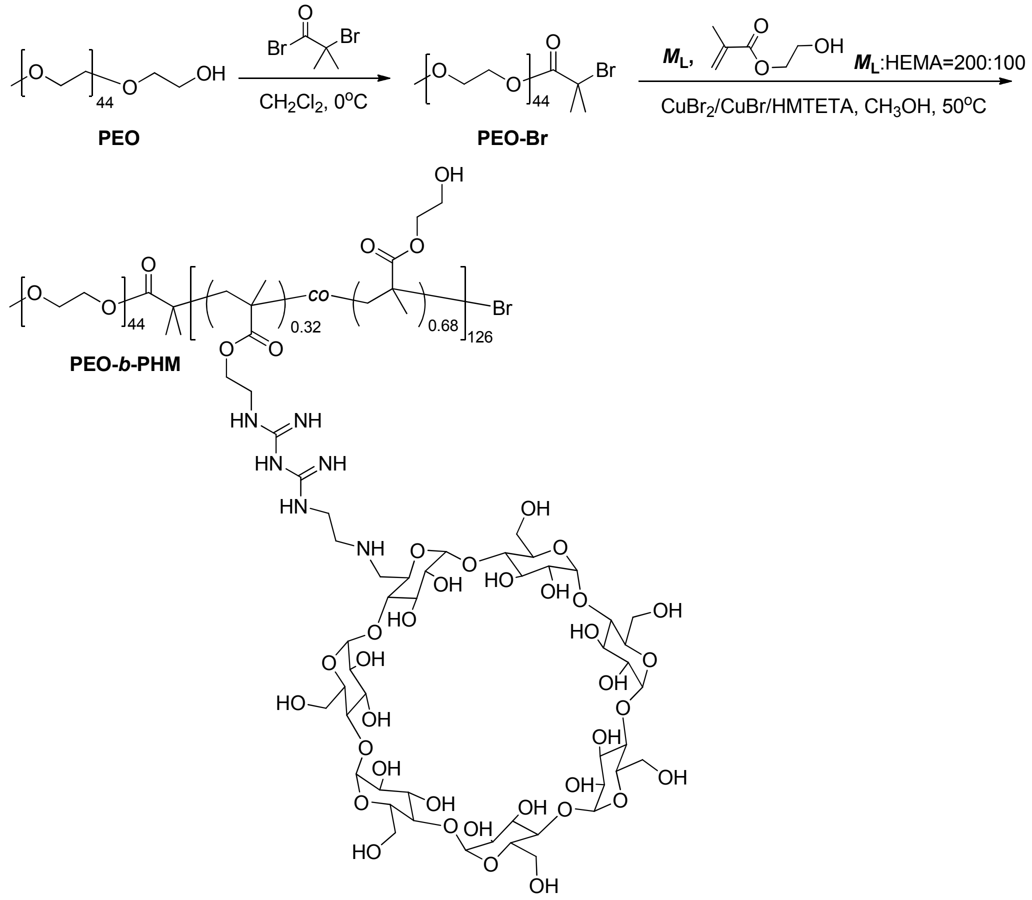

Fig. S3. The synthetic route of diblock copolymer PEO-*b*-PHM with bioinspired receptor pendants via ATRP protocol

Synthesis of poly(ethylene oxide)-based ATRP macromolecular initiator (PEO-Br). Mono-methoxy poly(ethylene oxide) (M_n = 2.0 kDa, M_w/M_n = 1.04, 2.02 g, 1.0 mmol) and triethylamine (0.10 g, 1.0 mmol) in 20 ml of CH_2Cl_2 at 0 °C, 2-bromo-isobutyryl bromide (0.28 g, 1.25 mmol) in 5 ml of CH_2Cl_2 was slowly injected dropwise for ~2 h. After the addition was completed, the mixture reacted for 24 h at room temperature under stirring. After the reaction, the resulting solution was washed with deionized water for three times to remove the water-soluble triethylamine hydrochloride. Then being dried with anhydrous sodium sulfate, the organic phase was concentrated to ~5 ml, and subsequently precipitated into 100 ml of ice diethyl ether for twice. Finally, the product was collected and dried in vacuum oven at 30 °C for 48 h, affording white solid (1.48 g, 69% yield). $M_{n,GPC}$ = 2400 g mol^{-1}, M_w/M_n = 1.09. ^{1}H NMR ($CDCl_3$, 300 MHz): δ_H (ppm) = 4.32 (s, -C***H$_2$***OOC-), 3.64 (s, -C***H$_2$***C***H$_2$***O-), 3.20 (s, -OC***H$_3$***), 1.95 (s, -OOCC(C***H$_3$***)$_2$Br)

Synthesis of diblock copolymer PEO-b-PHM via ATRP. The reaction was as follows[5]: the above PEO-Br macro-initiator (4 mg, 2 μmol), functionalized monomer $\boldsymbol{M}_{\mathbf{L}}$ (0.56 g, 0.4 mmol), 2-hydroxylethyl methacrylate (HEMA, 26 mg, 0.2 mmol), $CuBr_2$ (trace), CuBr (0.3 mg, 2 μmol), and 1,1,4,7,10,10-hexamethyltriehylenetetramine (HMTETA, 0.5 μl, 2 μmol) were added into round bottom flask and dissolved in 2 ml of methanol, followed by three freeze-pump-thaw cycles. The flask was reacted at 50 °C with stirring. After reaction for the given time (20 h), the resulting solution was immersed to liquid nitrogen in order to stop the free radical polymerization. Subsequently the solution was diluted to DMF and passed through a neutral alumina column twice to remove the copper catalysts. The organic filtrate was concentrated to ~10 ml, and then precipitated into 50 ml of cold diethyl ether for three times. The product was collected and dried in vacuum oven for 48 h, giving a white solid polymer PEO-*b*-PHM (0.14 g, conversion: ~20% for $\boldsymbol{M}_{\mathbf{L}}$). $M_{n,GPC}$ = 68500 g mol^{-1}, M_w/M_n = 1.26. ^{1}H NMR ($CDCl_3$, 300 MHz): δ_H (ppm) = 6.80 (s, -COOCH$_2$CH$_2$NH-C(=N***H***)-), 6.65 (s, -NH-C(=NH) NHC(=N***H***)-NH-), 5.65—5.79 (m, OH-2,3 in CD), 4.81 (m, H-1 in CD), 4.43—4.58 (m, OH-6 in CD), 4.30 (s, -COOC***H*₂**CH$_2$NH-), 4.14 (s, -COOC***H*₂**CH$_2$OH), 3.96 (s, -COOCH$_2$C***H*₂**OH), 3.52—3.75 (m, -C***H*₂**C***H*₂**O- in PEO, -COOCH$_2$C***H*₂**NH- and H-3,5,6 in CD), 3.2—3.45 (m, H-2,4 in CD), 2.82 (s, -NHC***H*₂**CH$_2$NH-), 2.64 (s, -NHCH$_2$C***H*₂**NH-), 1.89 (s, -C***H*₃**), 1.32—1.58 (m, -[C***H*₂**-C***H***(CH$_3$)]$_n$- in main chain).

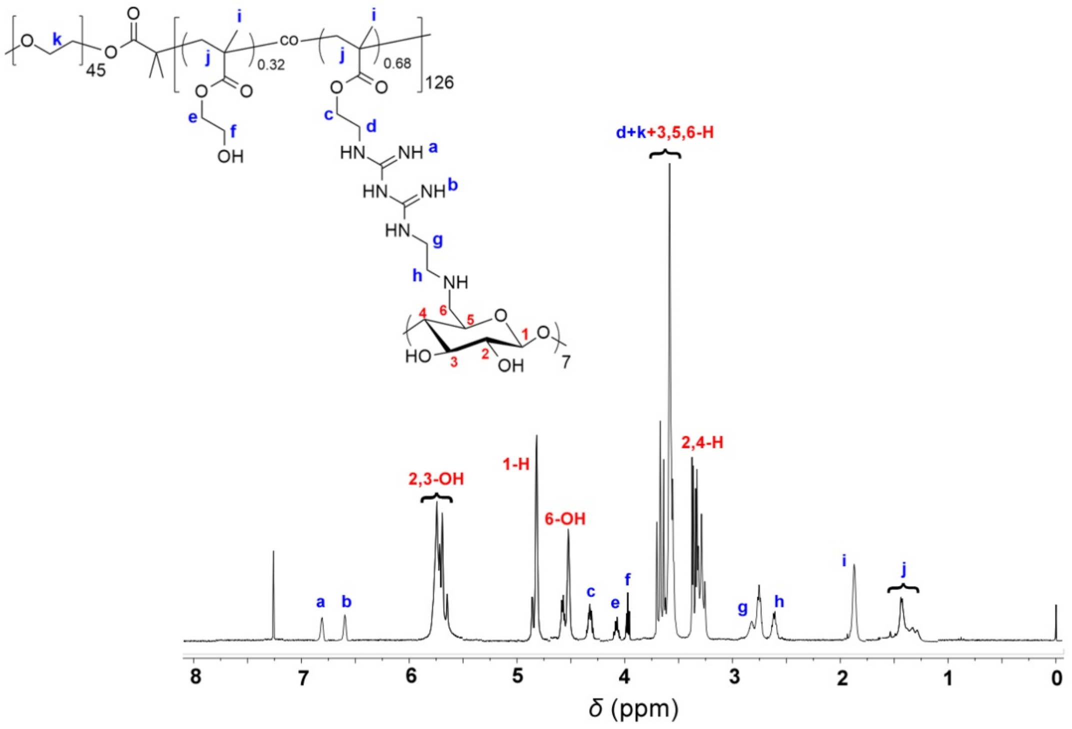

Fig. S4. ^{1}H NMR spectrum of the copolymer PEO-*b*-PHM

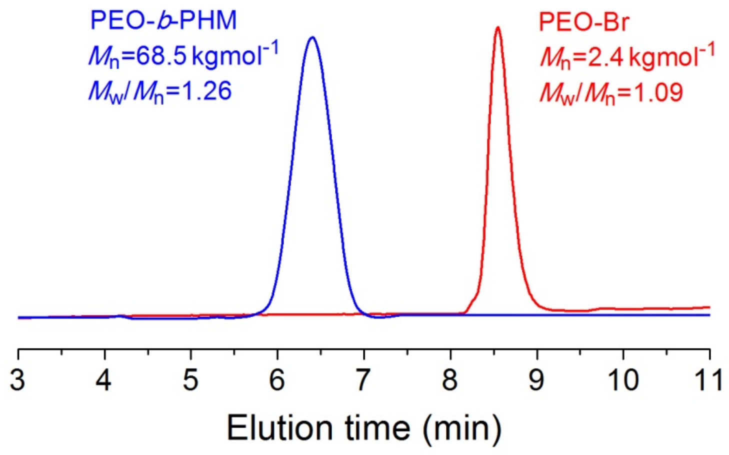

Fig. S5. GPC traces of PEO-Br macromolecular initiator and diblock copolymer PEO-*b*-PHM

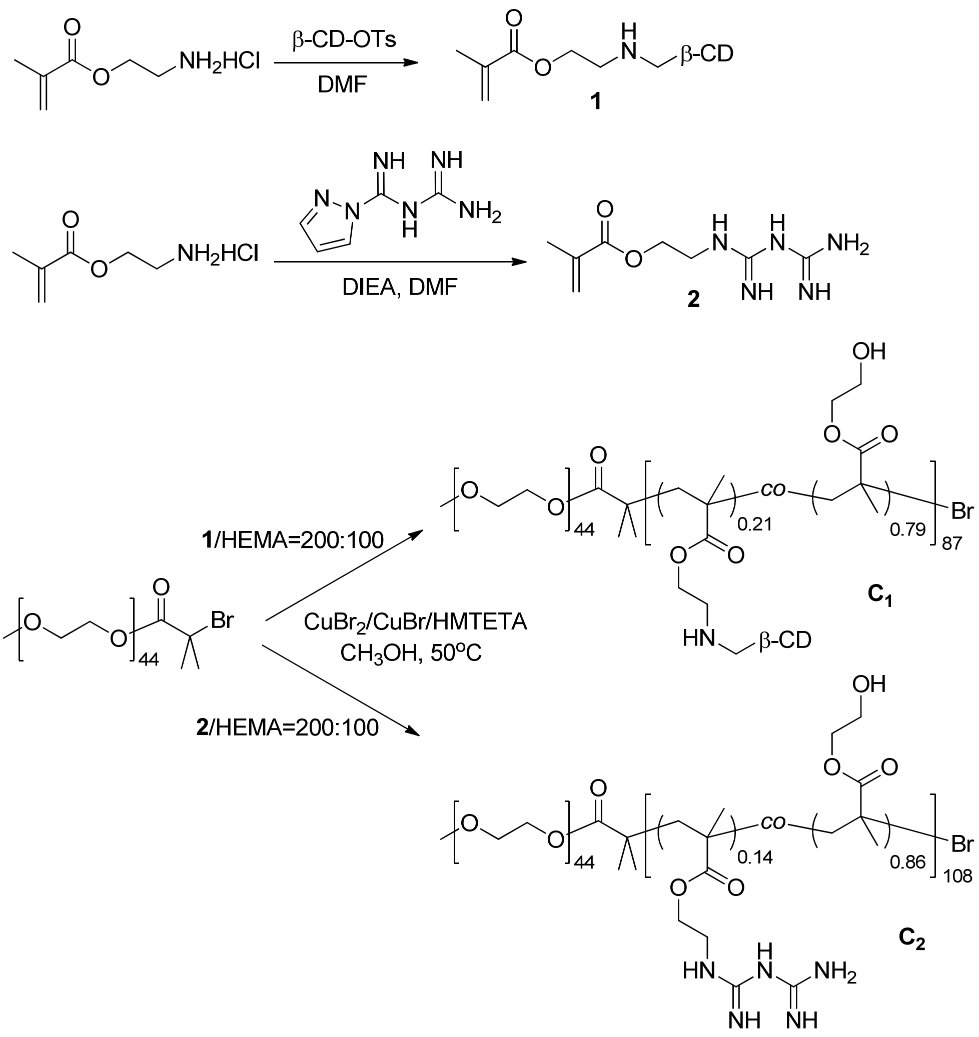

Fig. S6. The synthetic route of two copolymer counterparts (**C**$_1$ and **C**$_2$) via ATRP protocol

Synthesis of the copolymer counterparts C_1 and C_2. The synthetic procedures of $\mathbf{C_1}$ and $\mathbf{C_2}$ were both similar to that of PEO-*b*-PHM copolymer. The analogous monomer **1** (0.49 g, 0.4 mmol) and **2** (43 mg, 0.2 mmol), short of biguanidine spacer and β-CD pendant, was dissolved in 2 ml of methanol, respectively. By adding the same molar amounts of PEO-Br, CuBr, $CuBr_2$ and HMTETA to the solution, the reaction lasted for 16 h and stopped by immersion into liquid nitrogen. Subsequently the solution was diluted to DMF and passed through a neutral alumina column twice to remove copper catalysts. The organic filtrate was concentrated to ~10 ml, and then precipitated into 50 ml of cold diethyl ether for three times. $\mathbf{C_1}$ and $\mathbf{C_2}$ product was afforded in vacuum oven for 48 h.

2. ^{1}H-NMR and 2D-NOSEY Spectra for the Host-Guest Inclusion

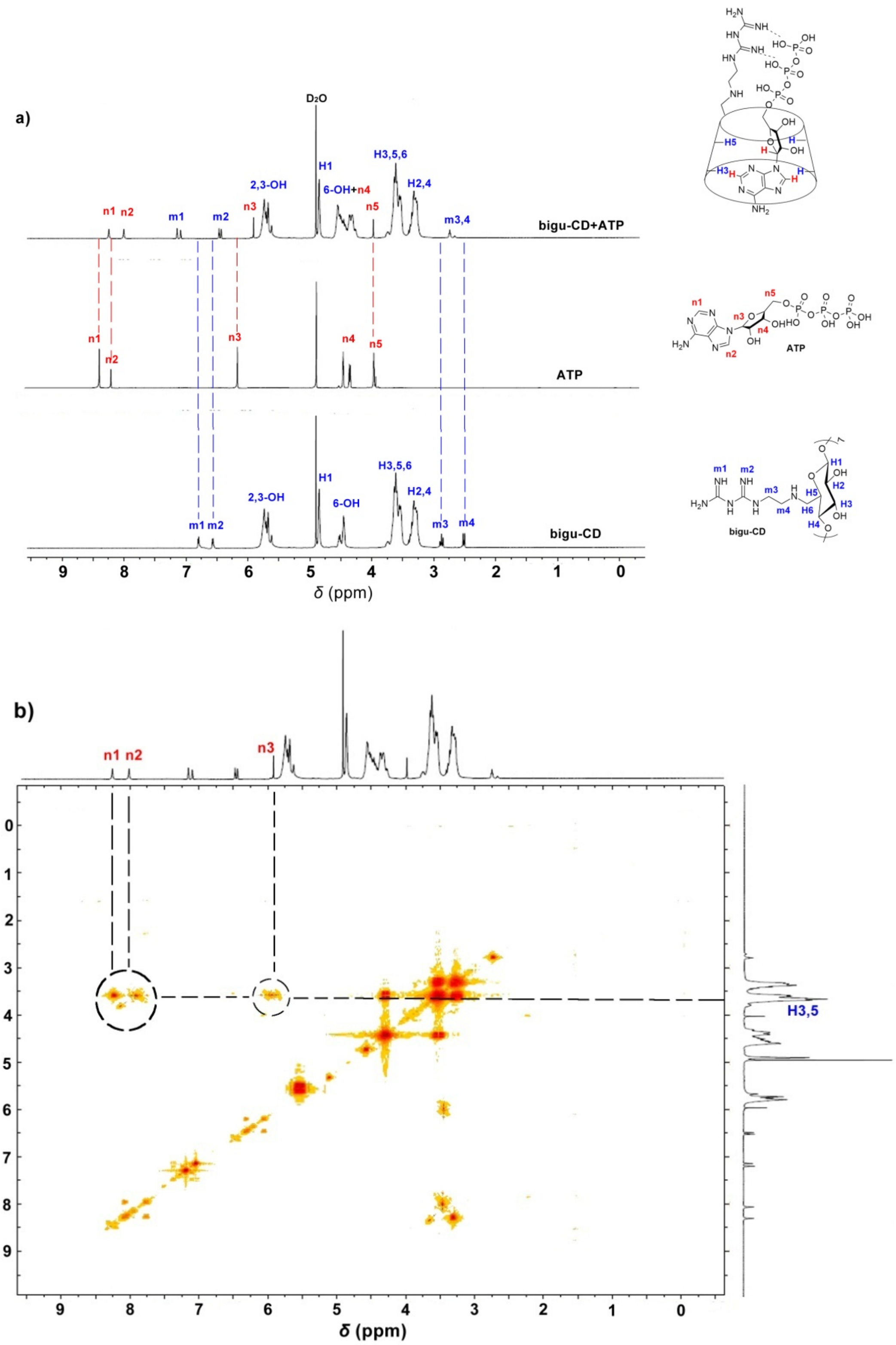

Fig. S7. (a) ^{1}H-NMR spectra (D_2O) of bigu-CD host (bottom), ATP guest molecule (middle), and bigu-CD-ATP (1:1 molar ratio). (b) 2D-NOSEY spectrum (D_2O) of bigu-CD-ATP complex (1:1 molar ratio).

Since there is lots of overlapping proton NMR signals between PEO-*b*-PHM polymer and ATP molecule (δ = 3.0~5.0), which makes the spectra complication and hard to distinguish, we chose β-cyclodextrin bearing a single biguanidine group (bigu-CD) as the model host unit. ^{1}H NMR spectroscopy was used to provide information on their host-guest interaction. As shown in **Fig. S7a**, independent ATP molecule showed its typical proton shifts in deuteroxide solvent at δ = 8.47 (n1), 8.25 (n2) and 6.23 (n3); the bigu-CD compound also exhibited its characteristic peaks at δ = 6.81 (m1), 6.57 (m2), 2.88 (m3) and 2.52 (m4). Upon formation of the host-guest inclusion complex, it was observed that these resonance of the protons on bigu-CD and ATP were shifted. The peaks ascribed to ATP guest has an upfield shift [δ = 8.47→8.28 (n1), 8.25→8.03 (n2) and 6.23→5.88 (n3)], but other signals such as n4 and n5 remain constant. This effect arises from the host-guest inclusion, thus strongly suggesting that the ATP guest is bound into the cavity of bigu-CD. The supramolecular arrangement and interacting orientation between ATP and bigu-CD was confirmed by 2D nuclear overhauser effect spectroscopy (2D-NOSEY), since the typical distance between the host and the guest is ranging from 3—5 Å, which is in the detectable range of NOE signals. As shown in **Fig. S7b**, the cross-correlation peaks of n1-H3, n2-H3 and n3-H3,5 were observed, which indicates that the adenosine group is trapped into the bigu-CD cavity near the bottom, and the ribosyl group is closed to the top faced to the H5 of bigu-CD moiety. Furthermore, from the proton shifts of m1 and m2 of the biguanidine group, it proves that the biguanidine group has H-bonding interaction to the triphosphate group of ATP guest.

3. Job's Plot for Measuring the Binding Sites

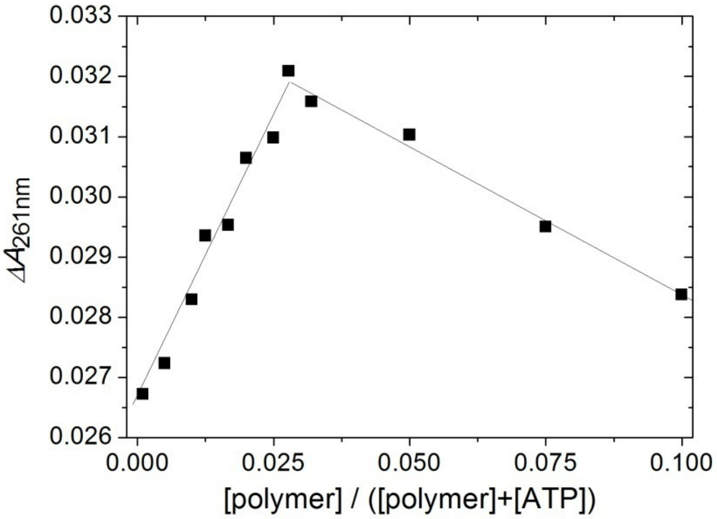

Fig. S8. Job's plot of the PEO-*b*-PHM and ATP (fixing the total concentration of PEO-*b*-PHM and ATP at 0.10 mM while varying the copolymer molar fraction from 0 to 0.10, the experiment is in Tris-HCl buffer, pH = 7.20)

To ensure the exact stoichiometry between PEO-*b*-PHM copolymer receptor and ATP bioactivator host-guest inclusion complex, we used UV-Vis spectra to supply a Job's plot experiment.[6] We fixed the total molar concentration of PEO-*b*-PHM and ATP at 0.10 mM, and then the molar ratio of the copolymer in the total concentration changes from 0 to 0.10. The intensity of UV-Vis absorption at 261 nm (ATP diagnostic peak) is recorded as the molar ratio of copolymer increases. From **Fig. S8**, the absorption reaches its maximum when the molar ratio is around 0.027, indicating that the binding stoichiometry between one PEO-*b*-PHM copolymer and ATP is about 36. This value is corresponding to the ITC result (binding site, n = 34.8).

4. ITC Data of ATP /PEO-*b*-PHM, ATP/C_1 and ATP/C_2

Table S1. ITC results of ATP binding with PEO-*b*-PHM and its two copolymer counterparts (Injecting 60 μM of ATP solution into 1.0 μM PEO-*b*-PHM, $\mathbf{C_1}$ and $\mathbf{C_2}$ solution at 293 K, respectively. All the experiments are in Tris-HCl buffer, pH = 7.20)

	**ATP**				
	K_B (M^{-1})	ΔH ($kJ\,mol^{-1}$)	ΔS ($J\,mol^{-1}K^{-1}$)	ΔG ($kJ\,mol^{-1}$)	n
**PEO-*b*-PHM**	7.52×10^7	-42.10	7.21	-44.20	34.8
$\mathbf{C_1}$	6.05×10^3	-19.20	6.62	-21.20	16.4
$\mathbf{C_2}$	1.42×10^3	-16.40	4.27	-17.7	3.9

5. Cooperative Effect of the Copolymer Receptor

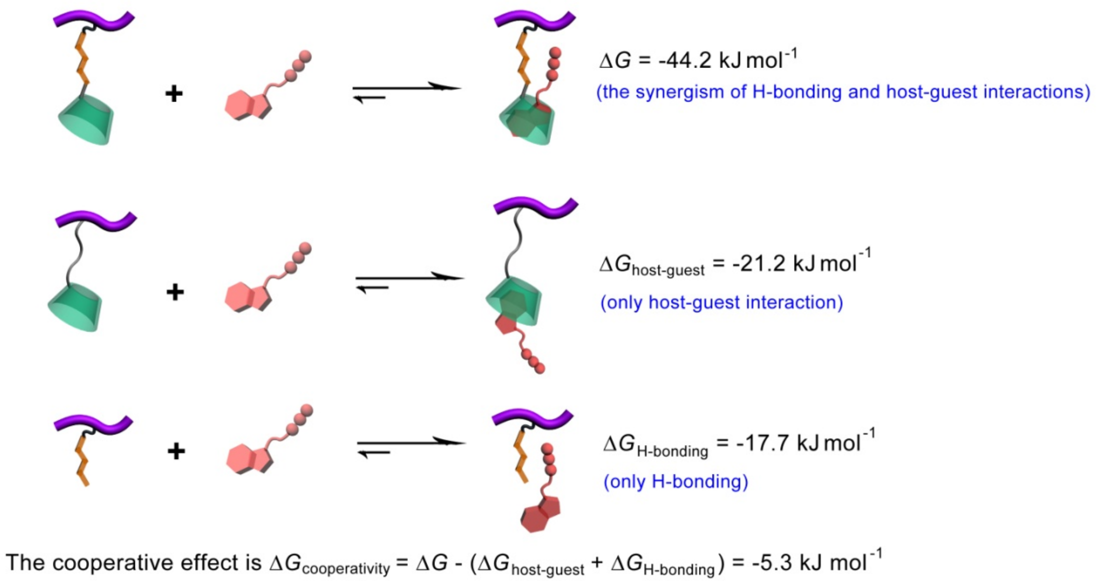

Fig. S9. The positive cooperativity of the PEO-*b*-PHM copolymer receptor binding to ATP biomolecule owing to the combination of GA and CD moiety

Based on the above discussion, the strong recognition of ATP biomolecule by our PEO-*b*-PHM copolymer receptor is relied on two kinds of supramolecular interactions: (i) H-bonding between the GA spacer in copolymer and the triphosphate species in ATP; (ii) host-guest interaction between the CD moiety in copolymer and the nucleoside in ATP. As shown in **Fig. S9**, the total Gibbs free energy including H-bonding and host-guest interaction (ATP/PEO-*b*-PHM) is -44.2 kJ mol^{-1} according to the top image. However, if we use $\mathbf{C_1}$ counterpart, lack of GA spacer, instead of PEO-*b*-PHM, the free energy falls down to -21.2 kJ mol^{-1}, indicating that the individual host-guest interaction is estimated to be -21.2 kJ mol^{-1} (middle image). In a similar way, if we use $\mathbf{C_2}$ that is lack of CD moiety, the individual H-bonding energy contribution can be determined to be -17.7 kJ mol^{-1} (bottom image). But the residual free energy (-5.3 kJ mol^{-1}) besides H-bonding and host-guest interaction should be the positive cooperative effect by combining the GA and CD moiety.[7]

6. Turbidity Changes of the Copolymer Solution upon ATP Stimulus

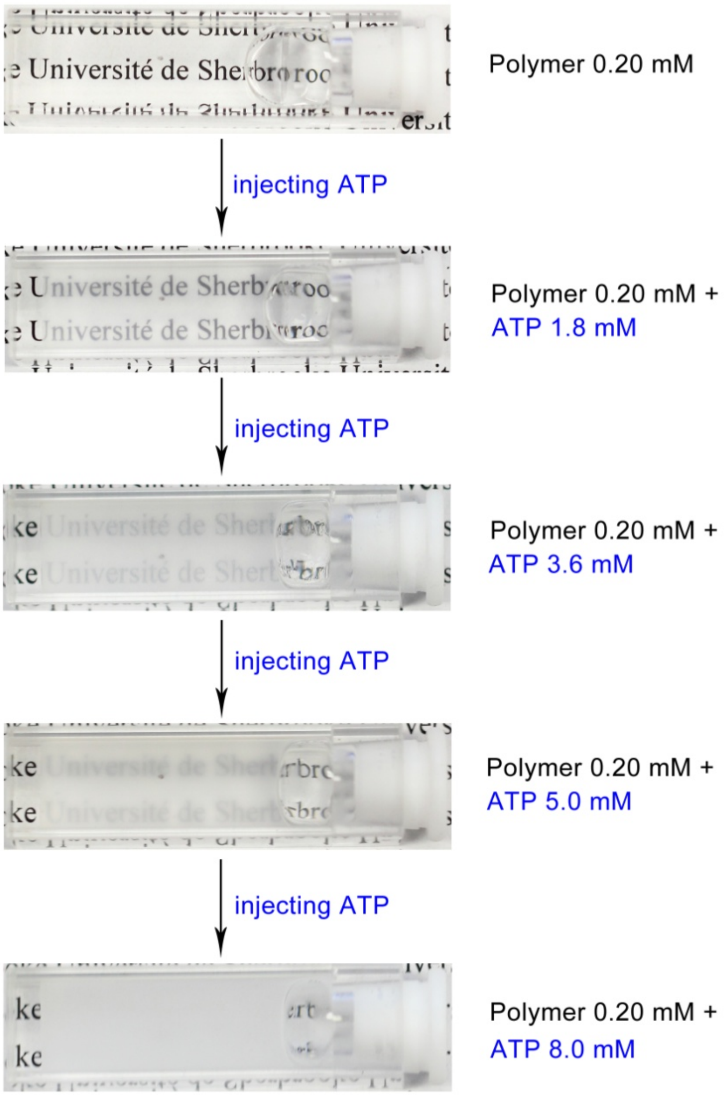

Fig. S10. The visualized turbidity variation of the PEO-*b*-PHM copolymer solution after addition of different concentrations of ATP stimulation (top to down, the added ATP concentration is 0, 1.8, 3.6, 5.0, and 8.0 mM, respectively

7. ATP/PEO-*b*-PHM Complex to Form Spherical Micelles

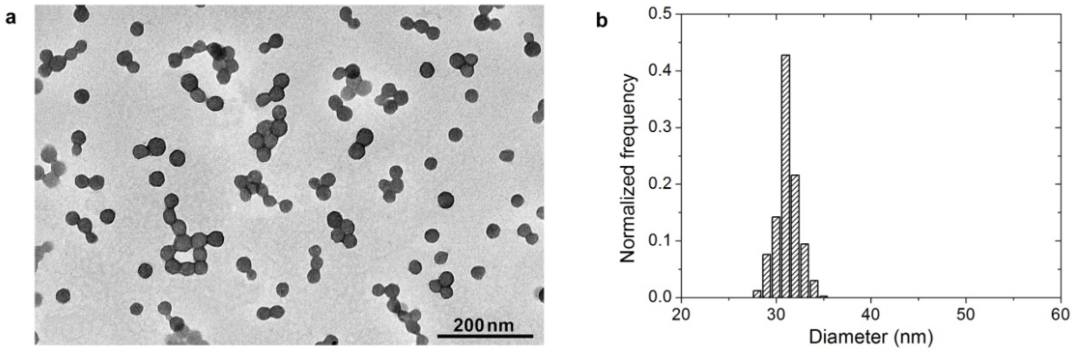

Fig. S11. (a) TEM image of the spherical micelles self-assembled by ATP/PEO-*b*-PHM complexes (PEO-*b*-PHM: 0.20 mM; ATP: 1.8 mM; ATP:PEO-*b*-PHM = 9:1). (b) Size distribution histogram from TEM statistics over 250 particles.

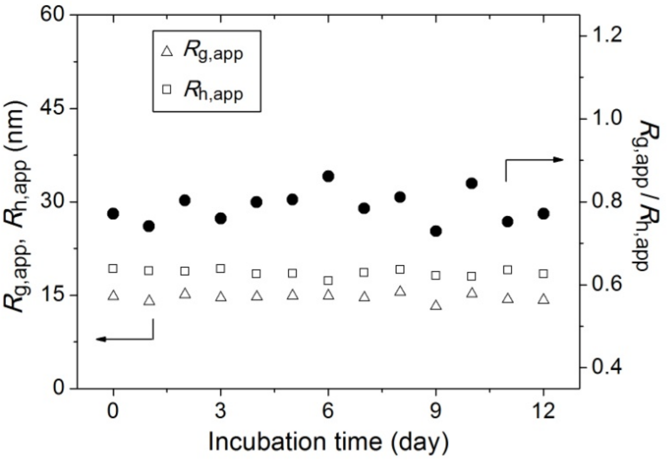

Fig. S12. The apparent gyration radius ($R_{g,app}$) measured by SLS, the hydrodynamic radius ($R_{h,app}$) measured by DLS, and the shape factor (ρ = R_g/R_h) changes of ATP/PEO-*b*-PHM spherical micellar aggregates plotted against incubation time. (The copolymer concentration is 0.20 mM and the ATP concentration is 1.8 mM, ATP:PEO-*b*-PHM = 9:1; the average ρ is calculated to be 0.789, corresponding to the theoretical value of solid sphere, $\rho_T = 0.774$.)

8. ATP/PEO-*b*-PHM Complex Deformation to Form Nanofibres

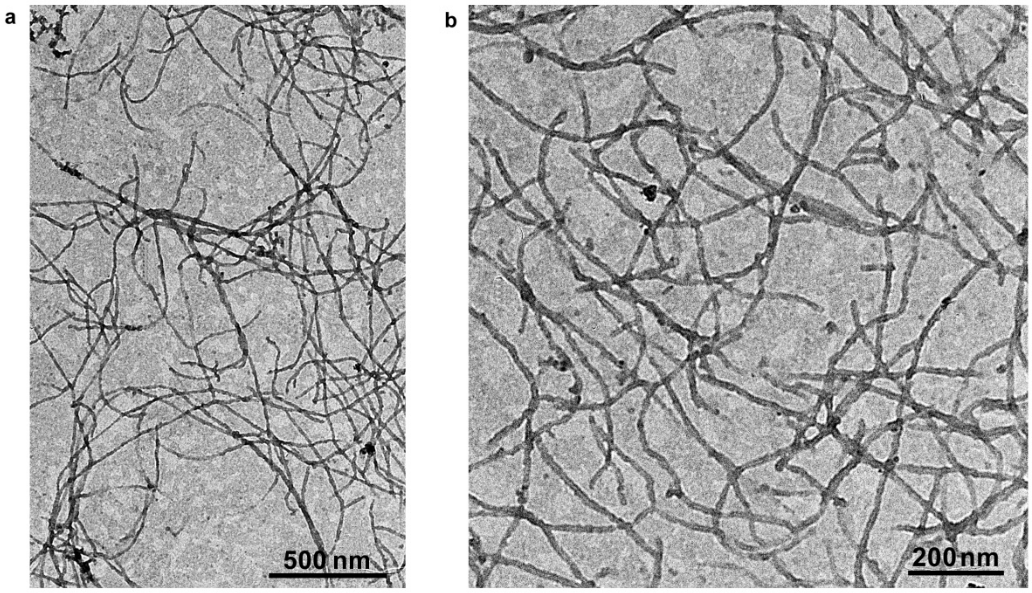

Fig. S13. (a) TEM image of the fibrous structure self-assembled by ATP/PEO-*b*-PHM complexes (PEO-*b*-PHM: 0.20 mM; ATP: 3.6 mM; ATP:PEO-*b*-PHM = 18:1). (b) Magnified TEM image of these ATP/polymer hybrid nanofibres.

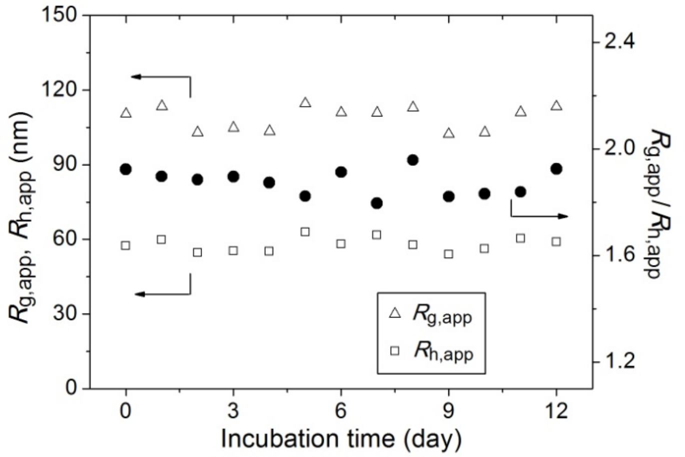

Fig. S14. The apparent gyration radius ($R_{g,app}$) measured by SLS, the hydrodynamic radius ($R_{h,app}$) measured by DLS, and the shape factor (ρ = R_g/R_h) changes of ATP/PEO-*b*-PHM nanofibrous aggregates plotted against incubation time. (The copolymer concentration is 0.20 mM and the ATP concentration is 3.6 mM, ATP:PEO-*b*-PHM = 18:1; the average ρ is calculated to be 1.893, corresponding to the theoretical value of cylinders, $\rho_T = 1.732$.)

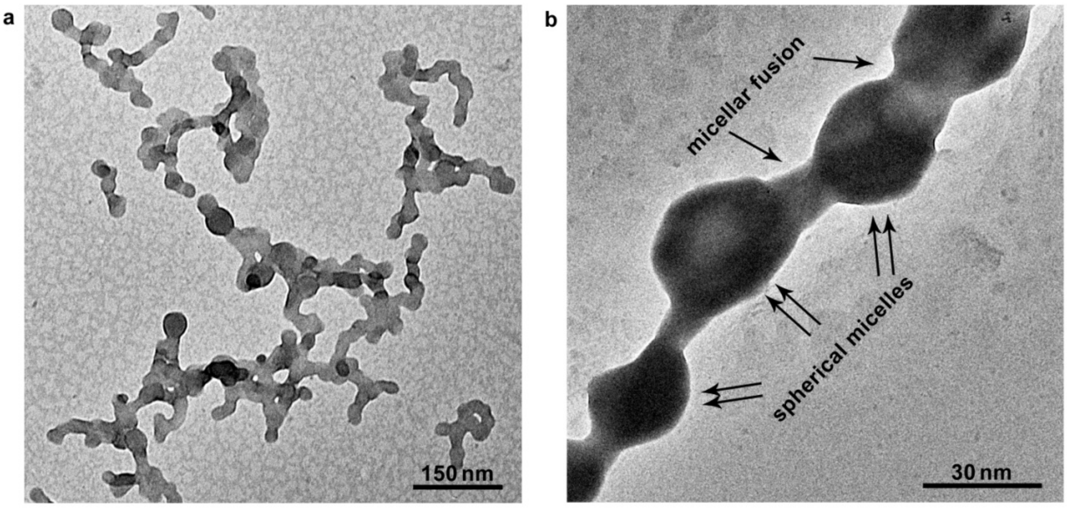

Fig. S15. (a) TEM images of the intermediate string-bead-like shape between spherical and fibrous morphology self-assembled by ATP/PEO-*b*-PHM complexes (PEO-*b*-PHM: 0.20 mM; ATP: 2.8 mM; ATP:PEO-*b*-PHM = 14:1). (b) Magnified TEM image of the string-bead-like structure to reveal the spherical micelle fusion mechanism to form ultralong nanofibres.

9. ATP/PEO-*b*-PHM Complex Deformation to Form Vesicles

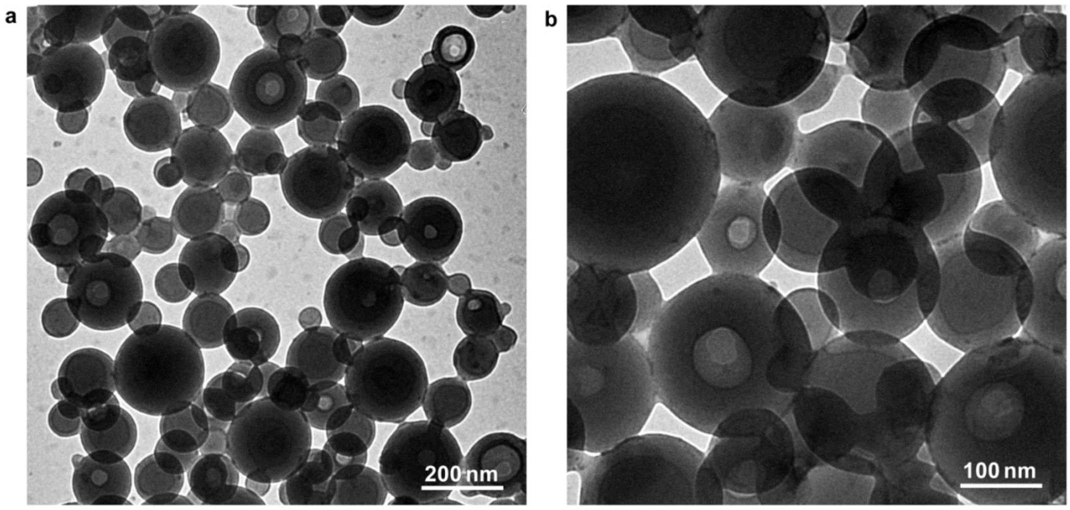

Fig. S16. (a) TEM image of the vesicular structure self-assembled by ATP/PEO-*b*-PHM complexes (PEO-*b*-PHM: 0.20 mM; ATP: 5.0 mM; ATP:PEO-*b*-PHM = 25:1). (b) The magnified TEM image of these ATP/polymer hybrid vesicular aggregates.

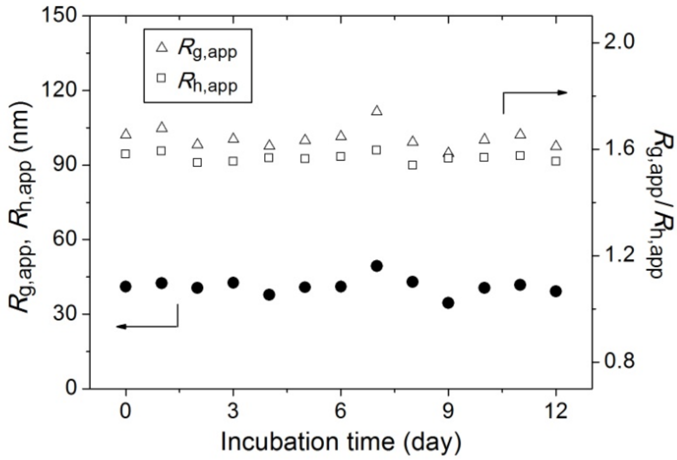

Fig. S17. The apparent gyration radius ($R_{g,app}$) measured by SLS, the hydrodynamic radius ($R_{h,app}$) measured by DLS, and the shape factor (ρ = R_g/R_h) changes of ATP/PEO-*b*-PHM vesicular aggregates plotted against the incubation time. (The copolymer concentration is 0.20 mM and the ATP concentration is 5.0 mM, ATP:PEO-*b*-PHM = 25:1; the average ρ is calculated to be 1.082, corresponding to the theoretical value of hollow spheres, $\rho_T \sim 1$.)

10. ATP/PEO-*b*-PHM Complex Deformation to Form Porous Sponge-Like Network Structures

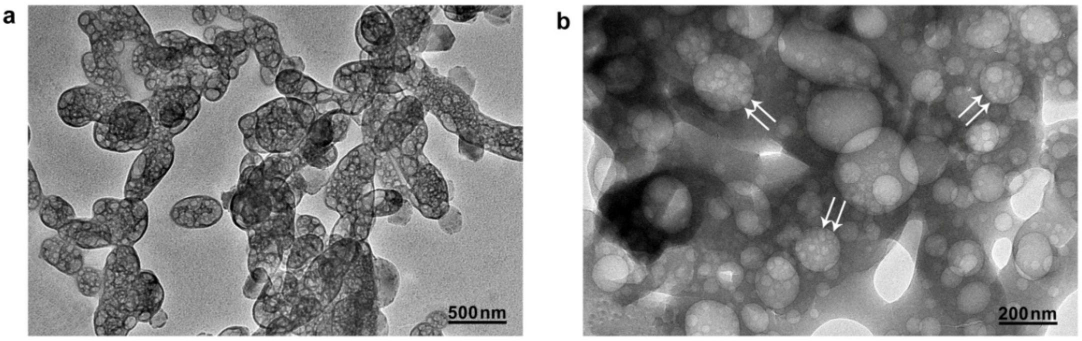

Fig. S18. (a) TEM image of the porous sponge-like network structure self-assembled by ATP/PEO-*b*-PHM complexes (PEO-*b*-PHM: 0.20 mM; ATP: 7.2 mM; ATP:PEO-*b*-PHM = 36:1). (b) The magnified TEM image of the porous architecture to indicate the vesicular interconnection and fusion mechanism. The size of these nanopores is ranging from 50 to 420 nm (white arrows), which is consistent with the diameter of the former vesicles.

11. ATP/PEO-*b*-PHM Complex Deformation to Form Interconnected Lamellar Structures

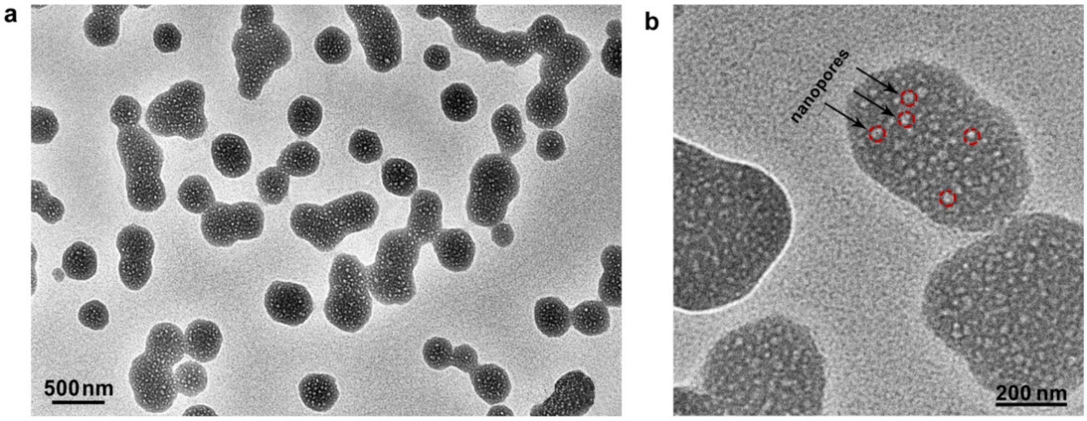

Fig. S19. (a) TEM image of the interconnected lamellar structure self-assembled by ATP/PEO-*b*-PHM complexes (PEO-*b*-PHM: 0.20 mM; ATP: 8.0 mM; ATP:PEO-*b*-PHM = 40:1). (b) The magnified TEM image of the porous channels in the lamellae to indicate the flatten process of the former porous networks. The size of the nanopores decreases to 20–40 nm (red circles), proving the flatten mechanism.

12. Molecular Weight Shift Triggered by ATP

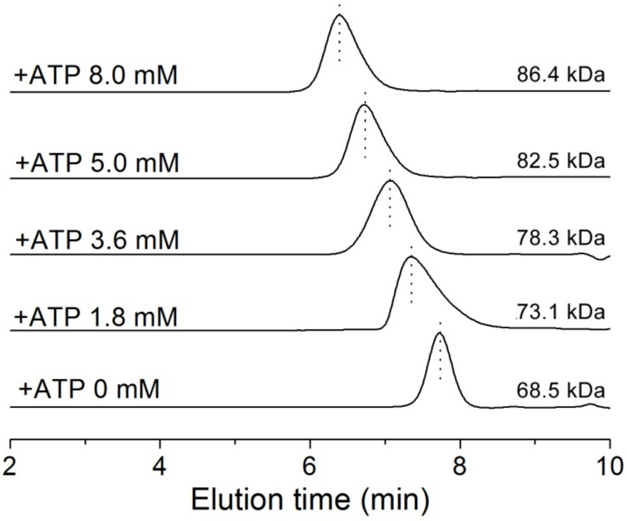

Fig. S20. GPC experiments showing the gradual molecular weight increase of the ATP/PEO-*b*-PHM complex after different concentrations of ATP treatment (0, 1.8, 3.6, 5.0, and 8.0 mM ATP, the copolymer concentration is fixed at 0.20 mM in Tris-HCl buffer, pH = 7.20)

Since the PEO-*b*-PHM is a type of block copolymer with polyvalent functional groups (binding site ≈ 36), one individual chain can seize quantitative ATP biomolecules in order to form the ligand-receptor complexes, further resulting in the self-assembly transition and morphological transformation. To monitor the amount of ATP captured by the copolymer chain, we utilize GPC to measure the alterations of the copolymer molecular weight upon ATP stimulation. Without any biomolecular stimulus, it shows the molecular weight of the PEO-*b*-PHM is ~68.5 kDa (**Fig. S20**). When ATP is added to the solution and reaches 1.8 mM, it can be captured by the copolymer chain and forms the ligand-receptor complex. GPC result reveals that the molecular weight of the complex increases up to 73.1 kDa. According to this result, we can calculate that per polymer chain has trapped n = 9.1 ATP biomolecules. With the increase of ATP concentration (1.8 mM→8.0 mM), the molecular weight gradually shifts from 73.1 kDa to 86.4 kDa. At the different ATP stimulation levels (0 mM, 1.8 mM, 3.6 mM, 5.0 mM and 8.0 mM), it is calculated that per polymer chain could capture 0, 9.1, 19.4, 27.6 and 35.2 ATP biomolecules, respectively, which indicates the ligand-receptor mechanism to drive the polymer shape transformation.

13. The Relationship between Hydrophilic Volume Fraction (*f*) and the Deformation of ATP/Polymer Complex Assemblies upon Different Levels of ATP Stimulus

Table S2. The relationship between calculated hydrophilic volume fraction parameter (*f*) and the experimental shape changes of the ATP/PEO-*b*-PHM complex assemblies at different levels of ATP stimulation

Entry	ATP stimulation concentration (mM)	association number *n*	ATP/polymer complex molecular weight (kDa)	*f*	shape	f_{theory}
0	0	0	68.5	100%	unimer	–
1	1.8	9.1	73.1	77%	sphere	>50%
2	3.6	19.4	78.3	52%	fibre	40%~50%
3	5.0	27.6	82.5	31%	vesicle	25%~40%
4	8.0	35.2	86.4	12%	porous lamella	<25%

According to the concept proposed by Discher et al., the shape of ATP/polymer aggregates can be dictated by the block hydrophilic-hydrophobic ratio. The change of hydrophilic volume fraction parameter (*f*)[8,9] reflects the variation of block copolymer amphiphilic aggregates to a certain extent. One can estimate the *f* value from the *Eq*.(1) as follows:

$$f = \frac{\sum V_{philic}}{\sum V_{total}} \times 100\% = \frac{\sum M_{philic}/\rho_{philic}}{\sum M_{philic}/\rho_{philic} + \sum M_{phobic}/\rho_{phobic}} \times 100\%$$

$$= (1 - \frac{\sum M_{phobic}/\rho_{phobic}}{\sum M_{philic}/\rho_{philic} + \sum M_{phobic}/\rho_{phobic}}) \times 100\% \text{..} (1)$$

Where the M_i is different block molar mass in the total molecular weight of the block copolymer, and ρ_i is the density of different blocks. The block copolymer are expected to form spherical micelles when $f < 50\%$, worm-like micelles when $40\% < f < 50\%$, vesicles for $25\% < f < 40\%$, and other complex lamellar structure for $f < 25\%$.

In our complexation systems (ATP/PEO-*b*-PHM), the contribution of the hydrophilic portion originates from the PEO, poly(2-hydroxyethyl methacrylate) block (PHMEA) and the non-complexation PHM block whereas the contribution of the hydrophobic portion is from the ligand-receptor ATP/polymer complexation block units. Thus, the *f* value in different levels of ATP stimulation can be calculated as *Eq*.(2):

$$f = (1 - \frac{n \times (M_L + M_{ATP})/\rho_{M_L}}{M_{PEO}/\rho_{PEO} + M_{PHMEA}/\rho_{HEMA} + MPHM/\rho_{M_L}}) \times 100\% \text{............................} (2)$$

where M_{PEO}, M_{PHEMA}, M_{PHM} and M_{ATP} are the different block molar molecular weight of PEO, PHEMA, PHM block and ATP biomolecule, respectively, and ρ_{PEO}, ρ_{HEMA}, ρ_{ML} are the density of PEO, PHEMA, and PHM block, and *n* is the association number of ATP and PEO-*b*-PHM which can be obtained from the above GPC results. The ρ_i is known from polymer handbook ($\rho_{PEO} = 1.091$ g cm^{-3}, $\rho_{HEMA} = 1.15$ g cm^{-3}, $\rho_{ML} = 1.76$ g cm^{-3},). The *f* value at different levels of ATP stimulus can be summarized in **Table S2**.

14. The Activity of Other ATP Biological Analogs

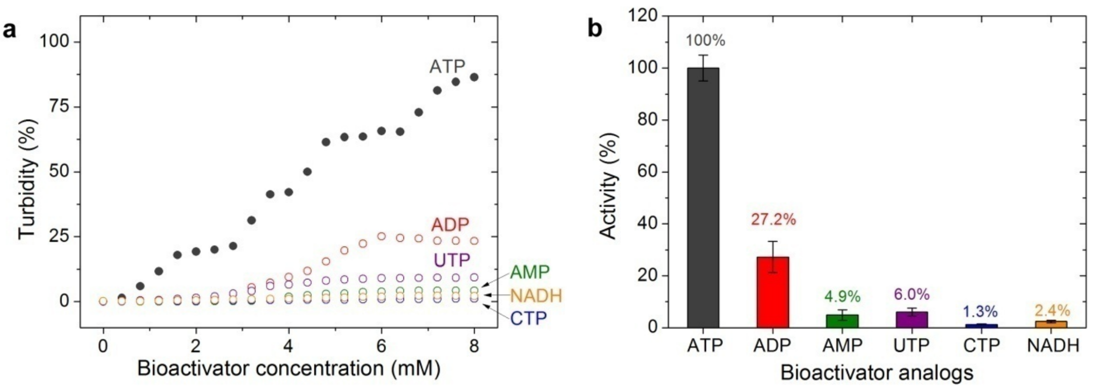

Fig. S21. (a) The turbidity variation of PEO-*b*-PHM copolymer solution upon various ATP biological analogs treatment in different stimulation level. (b) Histogram comparison of the activities of various ATP biological analogs. (The PEO-*b*-PHM concentration is fixed at 0.20 mM and the biological analogs' concentration gradually increases from 0 mM to 8.0 mM. All the experiments are carried out in the Tris-HCl buffer to keep the pH = 7.20)

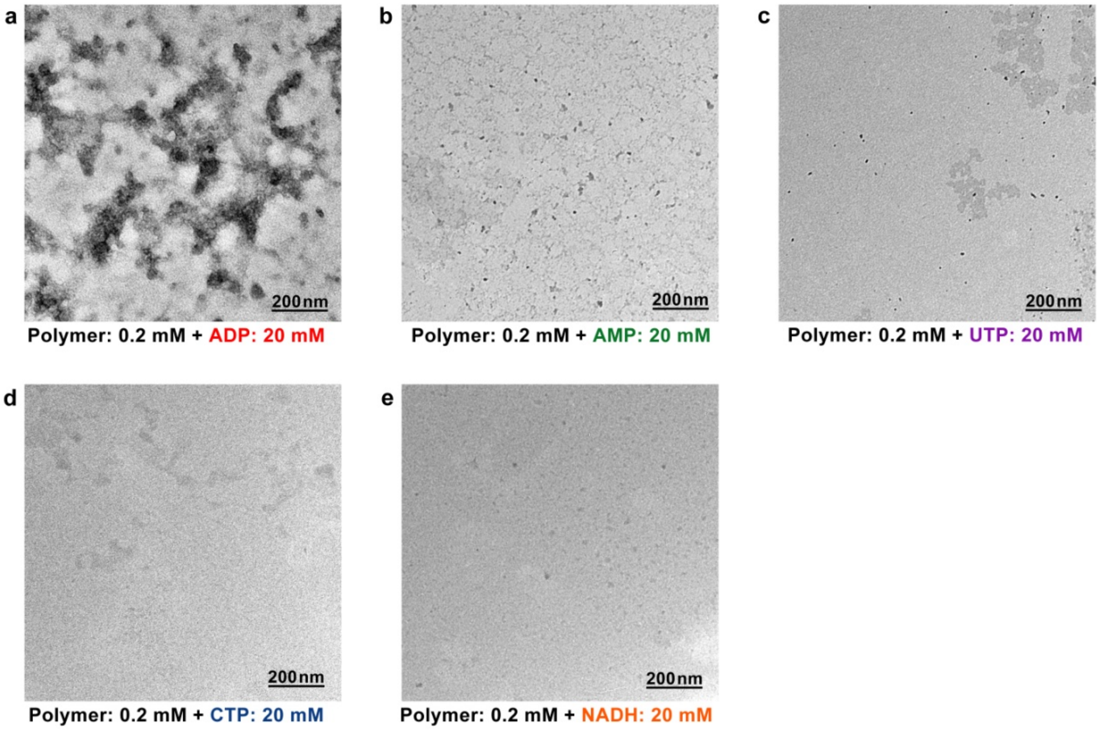

Fig. S22. TEM images of various ATP biological analogs (20 mM) injecting into the PEO-*b*-PHM copolymer solution (0.20 mM). (a) ADP, (b) AMP, (c) UTP, (d) CTP, (e) NADH.

To better suitable for applying in intracellular environment, it is desirable that our bio-responsive deformable polymer assemblies possess specific ATP activity. Because

once the bioactivator can trigger the polymer assembly, the turbidity of binary mixture should increase abruptly based on the aforementioned results. Thereby, we could take advantage of this character to survey whether some ATP biological analogs can drive a similar self-assembly and shape transformation. As shown in **Fig. S21**, except for ATP, other homologous bioactivators including ADP, AMP, UTP, CTP and NADH exhibit relatively low turbidity, indicating that these analogs are all unable to fuel the PEO-*b*-PHM copolymer to self-assemble into aggregates. If one regards the activity of ATP reference as 100%, the activities of other biological analogs to induce a similar deformation are extremely low (ADP: 27.2%, AMP: 4.9%, UTP: 6.0%, CTP: 1.3% and NADH: 2.4%). TEM images also demonstrate our viewpoint that there is no available aggregate formed when other biological analogs inject into the copolymer solution (**Fig. S22**).

15. Enzyme-Responsive Lamellar Disassembly

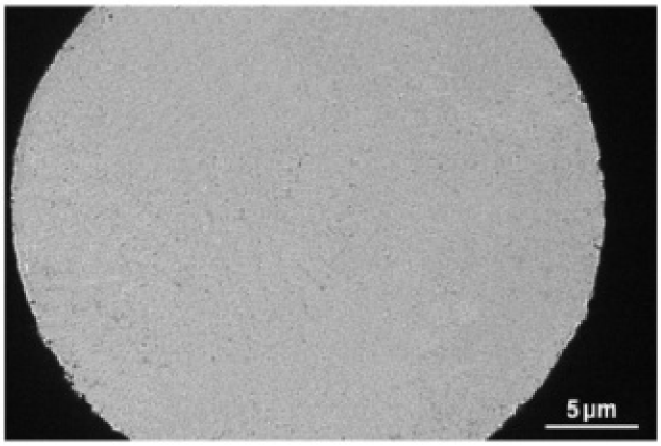

Fig. S23. TEM images of complete enzyme-responsive lamellar disassembly upon treatment with phosphatase for 6 h.

16. Method and Characterization

Sample preparation. The detailed synthesis and characterization of the copolymer PEO-*b*-PHM and its counterparts, C_1 and C_2, are described in the **Figure S1─S6**. The related chemical agents are bought from Sigma-Aldrich and Acros Ltd. Co. All the solvents are used as received.

ATP/PEO-*b*-PHM ligand-receptor complexation. In all self-assembly experiments, PEO-*b*-PHM copolymer is first dissolved in Tris-HCl buffer (pH = 7.20) to produce 0.20 mM aqueous solution (20 mL). Disodium salt of ATP (adenosine-5'-triphosphate disodium salt hydrate, M.W. = 551.14 g mol^{-1}) was dissolved in the Tris-HCl buffer solution (20 mL, pH = 7.20) at concentration of 0.2 M. Subsequently, the ATP solution was increasingly added into the above polymer solution under vigorous stirring through a micro-injector from 0 μL to 1000 μL at a rate of ~60 μL h^{-1}. From the beginning of the ATP/polymer complexation, the turbidity of the binary mixture was monitored at a fixed time interval (20 min). Meanwhile, we also took out aliquots of the hybrid assemblies solutions (0.2 mL) when the added amounts of ATP reached 80 μL, 180 μL, 360 μL, 500 μL, 720 μL, 800 μL and 1000 μL. Afterwards, these solutions (the molar ratios between ATP and polymer were 4:1, 9:1, 18:1, 25:1, 36:1, 40:1 and 50:1, respectively) were sealed in vials and incubated at 293 K for 24 hours before TEM characterization. All the experiments and characterization are carried out in the Tris-HCl buffer to keep the solution pH of 7.20.

Transmission electron microscopy. TEM imaging was performed on a Hitachi H-7500 microscope operating at an accelerating voltage of 120 kV. The TEM specimens were prepared by applying a drop of polymer solution (~5 μL) onto a carbon-coated copper TEM grid while allowing the solvent to evaporate quickly under freeze-drying for fixing the morphologies of these ATP/polymer hybrid assemblies. TEM images were collected on a Gatan CCD.

Laser light scattering. The DLS/SLS measurements were performed at a scattering angel of 90° on a Brookhaven (BI-200SM) equipped with a highly sensitive avalanche photodiode detector (Brookhaven, BI-APD), a digital correlator (Brookhaven, TurboCorr) that calculates the photon intensity autocorrelation function $g^2(t)$, and a helium-neon laser goniometer (λ = 632 nm). The PEO-*b*-PHM solution (0.20 mM) and all the binary ATP/polymer solutions (polymer at 0.20 mM while ATP is 1.8 mM, 3.6 mM, 5.0 mM and 7.2 mM, respectively) were filtered and then measured.

Spectroscopy. UV-Vis absorption spectra of the ATP/polymer complexes at different concentrations were measured using an Agilent Cary 6000i UV-Vis-NIR spectroscopy. All the samples were stirred for 2 hours and incubated at 293 K for 24 hours before measurement in order to make the ATP and PEO-*b*-PHM completely complexation. The transmittance of the polymer assemblies solutions with different amounts of ATP stimulation were monitored at 293 K by use of a Thermo Scientific (Varian Cary 50 Bio) UV-Vis spectrophotometer at a wavelength of 550 nm.

Isothermal titration calorimetry. ITC experiments were conducted using a Microcal

VP-ITC system at 293.15 K. The concentrations of the PEO-*b*-PHM and two counterparts C_1 and C_2 were all fixed at 1.0 μM for the injection of ATP aqueous solution (60 μM in Tris-HCl buffer, pH = 7.20). In a similar way, the ATP biological analogs (ADP, AMP, UTP, CTP, NADH) with the same concentration (60 μM) were injected into the PEO-*b*-PHM copolymer solution (1.0 μM) to compare the differences of their ligand-receptor binding affinity.

Nuclear magnetic resonance. ^{1}H, ^{13}C and ^{31}P NMR spectra were obtained by using a JEOL JNM-ECA 300 (300 MHz) spectrometer. The ^{1}H and ^{31}P-NMR titration was realized by slowly injecting the PEO-*b*-PHM solution to a fixed concentration of ATP solution in different molar ratios (1:400 → 1:10). The NMR solvent was chosen to be D_2O.

Modeling. The semiempirical calculations were performed using the Molecular Docking Simulation 2.1 software combined with SYSBL 7.3 to study the host-guest inclusion compounds. The molecular structures were generated by the molecular builder provided in the SYSBL 7.3 and optimized by means of the docking methods.

Gel permeation chromatography. GPC measurements were conducted on a system of multiangle laser light scattering. The system is equipped with a Waters degasser, a Waters 515 HPLC pump, a 717 automatic sample injector, a Wyatt Optilab DSP differential refractometer, and a Wyatt miniDAWN detector. Three chromatographic columns (PLgel mix-H: 7.5 × 300 mm, PLgel guard-H: 7.5 × 50 mm, and Shodex GPC KD-806M: 8 × 300 mm) were used in series. HPLC-grade dimethyl formamide (DMF) was used as eluent at a flow rate of 1.0 mL min^{-1} at 30 ^{o}C in the presence of LiBr (0.5 g L^{-1}). The ATP/PEO-*b*-PHM binary complexes can totally dissolve in DMF solution.

Fourier Transform Infrared Spectrometer. FT-IR analyses were performed on a Thermofisher NEXUS-470. The compounds were grinded and mixed with pure KBr powder to measure.

Mass Spectroscopy. Matrix-assisted laser desorption ionization time-of-flight (MALDI-TOF) mass spectra were obtained by using a SHIMADZU AXIMA-CFR Plus station. The matrix was chosen as dithranol.

References

(1) Lee, Y. L.; Martásek, P.; Roman, L. J.; Silverman, R. B. *Bioorg. Med. Chem. Lett.* **2000**, *10*, 2771-2774.

(2) Petter, R. C.; Salek, J. S.; Sikorski, C. T.; Kumaravel, G.; Lin, F. T. *J. Am. Chem. Soc.* **1990**, *112*, 3860-3868.

(3) Bernatowicz, M. S.; Wu, Y. L.; Matsueda, G. R. *J. Org. Chem.* **1992**, *57*, 2497-2502.

(4) Sibrian-Vazquez, M.; Nesterova, I. V.; Jensen, T. J.; Vicente G. H. *Bioconjugate Chem.* **2008**, *19*, 705-713.

(5) Matyjaszewski, K. *Macromolecules* **2012**, *45*, 4015-4039.

(6) Mohanty, J.; Bhasikuttan, A. C.; Nau, W. M.; Pal, H. *J. Phys. Chem. B.* **2006**, *110*, 5132-5138.
(7) Williamson, J. R. *Nat. Chem. Biol.* **2008**, *4*, 458-465.
(8) Discher, B. M.; Won, Y.-Y.; Ege, D. S.; Lee, J. C.-M.; Bates, F. S.; Discher, D. E.; Hammer, D. A. *Science* **1999**, *284*, 1143-1146.
(9) Discher, D. E.; Ahmed, F. *Annu. Rev. Biomed. Eng.* **2006**, *8*, 323-341.
